# De novo assembly, functional annotation, and analysis of the giant reed (*Arundo donax* L.) leaf transcriptome provide tools for the development of a biofuel feedstock

**DOI:** 10.1186/s13068-017-0828-7

**Published:** 2017-05-30

**Authors:** Chiara Evangelistella, Alessio Valentini, Riccardo Ludovisi, Andrea Firrincieli, Francesco Fabbrini, Simone Scalabrin, Federica Cattonaro, Michele Morgante, Giuseppe Scarascia Mugnozza, Joost J. B. Keurentjes, Antoine Harfouche

**Affiliations:** 1 Department for Innovation in Biological, Agro-food and Forest Systems, University of Tuscia, Via S. Camillo de Lellis snc, 01100 Viterbo, Italy; 2 Alasia Franco Vivai s.s., Strada Solerette, 5/A, 12038 Savigliano, Italy; 3 IGA Technology Services, Via J. Linussio, 51-Z.I.U, 33100 Udine, Italy; 4 Department of Agricultural and Environmental Sciences, University of Udine, Via delle Scienze, 206, 33100 Udine, Italy; 5 Institute of Applied Genomics, Via J. Linussio, 51-Z.I.U, 33100 Udine, Italy; 6 Laboratory of Genetics, Wageningen University, Droevendaalsesteeg 1, 6708 PB Wageningen, The Netherlands

**Keywords:** Biofuel, De novo leaf transcriptome, RNA-Seq, Genic-SSRs, Phenylpropanoid, Purine and thiamine metabolism, Carbon fixation, Stomata, SAPs, *Arundo donax*

## Abstract

**Background:**

*Arundo donax* has attracted renewed interest as a potential candidate energy crop for use in biomass-to-liquid fuel conversion processes and biorefineries. This is due to its high productivity, adaptability to marginal land conditions, and suitability for biofuel and biomaterial production. Despite its importance, the genomic resources currently available for supporting the improvement of this species are still limited.

**Results:**

We used RNA sequencing (RNA-Seq) to de novo assemble and characterize the *A. donax* leaf transcriptome. The sequencing generated 1249 million clean reads that were assembled using single-*k*-*mer* and multi-*k*-*mer* approaches into 62,596 unique sequences (unitranscripts) with an N50 of 1134 bp. TransDecoder and Trinotate software suites were used to obtain putative coding sequences and annotate them by mapping to UniProtKB/Swiss-Prot and UniRef90 databases, searching for known transcripts, proteins, protein domains, and signal peptides. Furthermore, the unitranscripts were annotated by mapping them to the NCBI non-redundant, GO and KEGG pathway databases using Blast2GO. The transcriptome was also characterized by BLAST searches to investigate homologous transcripts of key genes involved in important metabolic pathways, such as lignin, cellulose, purine, and thiamine biosynthesis and carbon fixation. Moreover, a set of homologous transcripts of key genes involved in stomatal development and of genes coding for stress-associated proteins (SAPs) were identified. Additionally, 8364 simple sequence repeat (SSR) markers were identified and surveyed. SSRs appeared more abundant in non-coding regions (63.18%) than in coding regions (36.82%). This SSR dataset represents the first marker catalogue of *A. donax*. 53 SSRs (PolySSRs) were then predicted to be polymorphic between ecotype-specific assemblies, suggesting genetic variability in the studied ecotypes.

**Conclusions:**

This study provides the first publicly available leaf transcriptome for the *A. donax* bioenergy crop. The functional annotation and characterization of the transcriptome will be highly useful for providing insight into the molecular mechanisms underlying its extreme adaptability. The identification of homologous transcripts involved in key metabolic pathways offers a platform for directing future efforts in genetic improvement of this species. Finally, the identified SSRs will facilitate the harnessing of untapped genetic diversity. This transcriptome should be of value to ongoing functional genomics and genetic studies in this crop of paramount economic importance.

**Electronic supplementary material:**

The online version of this article (doi:10.1186/s13068-017-0828-7) contains supplementary material, which is available to authorized users.

## Background

Giant reed (*Arundo donax* L.) is a polyploid perennial rhizomatous grass, belonging to the Poaceae family. Although the debate about its origin is still outstanding, recently a Middle-East provenance of *A. donax* has been suggested [1]. *A. donax* is recognized as one of the most promising lignocellulosic crops for the Mediterranean area, due to its high biomass yield, low irrigation and nitrogen input requirements, and excellent drought tolerance. Moreover, *A. donax* biomass feedstock can be readily converted to heat and/or electricity and can also be processed to produce biofuels and biomaterials [2]. Because of these advantages, *A. donax* is expected to play a major role in the provision of lignocellulosic biomass across much of Europe.

Even though *A. donax* can produce panicle-like flowers, no viable seed production for Mediterranean ecotypes has been ever reported [3]. Consequently, there has been little agronomic improvement in this species. The rapid development of next-generation sequencing (NGS) technology provides us an opportunity to develop novel genomic tools and enable rapid improvement of *A. donax*. RNA sequencing (RNA-Seq) has been successfully and increasingly used to define the transcriptome in plants [4–8]. Transcriptome sequencing by RNA-Seq also holds great potential as a platform for molecular breeding and the generation of molecular markers [9]. De novo RNA-Seq assembly facilitates the study of transcriptomes for non-model plant species without sequenced genomes by enabling an almost exhaustive survey of their transcriptomes and allowing the discovery of virtually all expressed genes in a plant tissue. It also helps reconstruct transcript sequences from RNA-Seq reads without the aid of genome sequence information.

Despite its economic importance, genomic sequence resources available for *A. donax* are limited. Currently, a Blast web server was implemented to search for homologs of known genes in a transcriptome of bud, leaf, culm, and root tissues of one ecotype [10]. This database was further enriched with transcripts from shoot and root tissues of young plants grown in a growth chamber under polyethylene glycol (PEG)-induced osmotic/water stress [11]. Moreover, a shoot transcriptome of an invasive ecotype representing high and low confidence transcripts is accessible at the Transcriptome Shotgun Assembly (TSA) sequence database [12]. Genotype- and developmental stage-specific transcriptomes [13–15], generated from multiple individuals within a species, from different geographical origins, at different growth stages and subjected to environmental stresses under natural field condition, will ultimately lead to a comprehensive catalogue of gene expression. Therefore, using different ecotypes of *A. donax*, treatments, and time points/developmental stages is urgently needed to provide a more complete gene expression catalogue and allow a comprehensive comparison among various assemblies. Here we describe the genomic resources that we have generated using three ecotypes originating from distant geographical locations. We provide information on transcriptomic data with (a) improved coverage across ecotypes and drought stress conditions, (b) in-depth transcriptome assembly quality assessments, and (c) improved annotations, which will enrich the available data for *A. donax* and make these datasets directly valuable to other researchers.

To date, there are no genetic markers reported for *A. donax*. Simple sequence repeats (SSRs) or microsatellites are an ideal choice for developing markers for *A. donax* because of their abundance, high polymorphism, codominance, reproducibility, and cross-species transferability. Exploiting transcriptome data for the development and characterization of gene-based SSR markers is of paramount importance. In contrast to genomic SSRs, genic-SSRs are located in the coding region of the genome, developed in a relatively easy and inexpensive way, and are highly transferable to related taxa [16]. Therefore, they can directly influence phenotype and also be in close proximity to genetic variation in coding or regulatory regions corresponding to traits of interest. SSRs located in coding and untranslated regions can be efficient functional markers [17]. Despite their advantages, no SSR markers for *A. donax* are currently available.

Here we report the sequencing, de novo assembly, and annotation of the leaf transcriptome of *A. donax*, as well as the development of polymorphic and functional genic-SSR markers. RNA was extracted from leaf tissues of three ecotypes of *A. donax* grown under two water regimes in field conditions. Illumina sequencing produced 1252 million RNA-Seq reads and 1249 million clean reads were used for the de novo transcriptome assembly. The leaf transcriptome assembly consists of 62,596 unitranscripts with a mean length of 842 bp and a total nucleotide count of about 52.7 megabase pair (Mbp). The high-quality unitranscripts selected through the EvidentialGene pipeline were further analyzed for Benchmarking Sets of Universal Single-Copy Orthologues (BUSCOs) to assess the completeness of the transcriptome assembly and gene prediction. A global comparison of homology between the transcriptomes of *A. donax* and four other species of the Poaceae family revealed a high level of global sequence similarity within this family. The assembly was functionally annotated using several public databases: the Swiss-Prot section of the UniProt KnowledgeBase (UniProtKB/Swiss-Prot), UniProt reference clusters (UniRef90), NCBI (National Center for Biotechnology Information) non-redundant (NR) database, Gene Ontology (GO), and Kyoto Encyclopedia of Genes and Genomes (KEGG). In addition, comparative analyses with several phylogenetically related species with more complete genomic information were performed, allowing the identification of putative genes controlling important agronomic or domestication traits. Particularly, genes encoding for stress-associated proteins (SAPs), for lignin and cellulose biosynthesis, purine and thiamine metabolism, and stomatal development and distribution were analyzed in the leaf transcriptome. The identification of homologs of these genes leads to supportable hypotheses of their conserved functions and to reasonable strategies for their use in *A. donax* genetic improvement. Additionally, simple sequence repeats (SSRs) were identified in the leaf transcriptome, thereby obtaining the first genetic marker catalogue for *A. donax*, which could be used for population genetics studies. This new transcript dataset provides the most comprehensive resource currently publicly available for gene expression and gene discovery in *A. donax*, and the SSR markers developed in this study will facilitate further genetic and genomic research in *A. donax* and related plant species. The leaf transcriptome developed in this study is made available in LabArchives at doi:10.6070/H4W37TC2. All the Illumina sequencing reads generated in this study are deposited in the NCBI SRA (PRJNA360076). The SSR markers developed based on de novo transcriptome assembly are also included in Additional files 1, 2, 3, 4, 5, 6, 7, 8, 9, and 10.

## Methods

### Plant material, experimental design, and RNA isolation

Three ecotypes of *A. donax* named EcoA, EcoB, and EcoC were selected from a panel of 81 ecotypes collected along the Mediterranean basin for this study because of their great biomass yield potential and contrasting responses to drought stress. EcoA originates from a coastal habitat of Greece that is characterized by hot and dry climatic conditions, whereas EcoB from a coastal habitat of Croatia that is considered a transitional zone between subtropical and semiarid climates, and EcoC from an hilly habitat in northern Portugal that is characterized by a transitional zone between Mediterranean subtropical and European oceanic climates.

In 2014, six replicate plots of each selected ecotype were planted in Savigliano (SAV, 44°35′N, 07°37′E, 349 m above sea level), northern Italy, in a plot-scale experimental design using homogeneous rhizome cuttings. Each plot measured 10 m^2^ (2.5 m × 4.0 m) and contained 30 rhizomes planted at a distance of 0.5 m × 1.0 m. During this first year of growth, no serious insect infestation or diseases were observed, and no pesticide or fungicide was used. Plants were irrigated until field capacity and then grown under two watering regimes during the dry season: well-watered (WW) and natural moderate drought stress (mDr). The mDr was gradually imposed by decreasing the water availability from 80% to approximately 40% of field capacity, whereas WW plants, used as control, were maintained to approximately 80% of field capacity with drip irrigation to replenish evapotranspiration losses. Soil volumetric water content was continuously checked in the two treatments and was maintained near the field capacity in WW. Additionally, daily water deficit was calculated as the difference between the water availability (i.e., rainfall) and the crop water demand (i.e., specific crop evapotranspiration). A detailed description of drought treatments, soil water status, and meteorological conditions is shown in Additional files 1 and 2: Figure S1. The whole experiment lasted 73 days, during which three samplings, designated as T1 (DOY 198), T2 (DOY 218), and T3 (DOY 248), corresponding to 23, 43, and 73 days after water stress imposition, were performed for leaves.

Fully expanded, non-senescing leaves (the 5th from the top) were harvested at the three time points (T1, T2, and T3), immediately frozen in liquid nitrogen, and stored at −80 °C until further processing. A total of fifty-four leaf samples (3 ecotypes × 2 treatments × 3 biological replicates × 3 time points) were then ground in liquid nitrogen with precooled mortars and pestles. These samples are envisaged to represent a large proportion of the total leaf transcriptome in *A. donax* during the exponential growth phase in different environmental conditions. Total RNA was extracted from 50 mg of ground tissue using a Spectrum™ Plant Total RNA Extraction Kit (Sigma-Aldrich, St. Louis, MO) including DNase I treatment with an On-Column DNase I Digestion Set (Sigma-Aldrich, St. Louis, MO) according to the manufacturer’s instructions. Purity of total RNA was determined by spectrophotometric analysis using a T60 UV/VIS spectrophotometer (PG Instruments, Leicestershire, United Kingdom). RNA samples with 260/280 and 260/230 ratios ranging from 1.8 to 2.2 were accepted. The integrity of extracted RNA was visually checked by 1.2% (w/v) agarose gel electrophoresis. The ethidium bromide-stained gels were photographed using a Kodak-DC290 zoom camera and analyzed with the Kodak 1D Image Imaging Analysis Software (Eastman Kodak Company, Rochester, NY). The quantity of total RNA was determined with a Qubit^®^ 2.0 Fluorometer using the Qubit^®^ RNA BR assay kit (Invitrogen™).

### cDNA library construction and Illumina sequencing

The fifty-four total RNA samples from the three *A. donax* ecotypes were used for the construction of cDNA libraries and for Illumina sequencing reactions. Total RNA integrity was further checked using a Caliper GX (PerkinElmer, Waltham, MA). 1.5 µg of good-quality RNA was used as input of the ‘TruSeq Stranded mRNA Sample Prep kit’ (Illumina, San Diego, CA) for library preparation following the manufacturer’s instructions. Briefly, the poly-A containing mRNA in each of the samples was purified and enriched using magnetic oligo(dT)-rich beads, and then fragmented using divalent cations at high temperature. Next, cDNA was synthesized by reverse transcription and standard blunt-ending plus add ‘A.’ Illumina TruSeq adapters with indexes were ligated to the ends of the cDNA fragments. After ligation reaction and separation of non-ligated adapters, samples were amplified by PCR to selectively enrich the cDNA fragments in the library that have adapters at both ends. To ensure that the quality of the libraries was appropriate for sequencing, the concentration and insert size of final libraries were determined using a Qubit 2.0 Fluorometer (Invitrogen, Carlsbad, CA) and an Agilent 2100 Bioanalyzer High Sensitivity and DNA 1000 assay (Agilent Technologies, Santa Clara, CA). Finally, cDNA libraries were processed with Illumina cBot for cluster generation on the flow cell following the manufacturer’s instructions and sequenced in single-end mode using a HiSeq2500 sequencing platform (Illumina, San Diego, CA). The CASAVA v1.8.2 of the Illumina pipeline was used to process raw data for format conversion and de-multiplexing. On average, 23 million 50-bp reads were produced for each sample.

### Quality control and filtering of read dataset

Reads were processed for quality assessment and removal of low-quality bases before de novo assembly of a leaf transcript catalogue. Subsequently, quality control of raw reads was performed using FastQC v0.11.3 (available at http://www.bioinformatics.babraham.ac.uk/projects/fastqc/) [18] and a quality score above Q30 was maintained in all fastq files for downstream analysis. We further excluded reads containing at least one undefined (N) base using an inhouse-built script in Python v2.7. Finally, we used FASTX-Collapser (downloaded from http://hannonlab.cshl.edu/fastx_toolkit/index.html) [19] to collapse identical sequences from fastq files of each biological replicate in the two conditions and in each time point into a single file, while maintaining read counts. Hence, a comprehensive dataset, representing a large proportion of *A. donax* leaf transcriptome, was generated into a single fasta file, which was used for the subsequent assembly step.

### De novo assembly strategy for leaf transcriptome reconstruction

In our assembly pipeline, the de novo leaf transcriptome reconstruction was performed using a two-step procedure. The first step (hereafter termed pre-assembly step) was carried out using two different approaches in order to capture a maximum number of transcripts. These two approaches employed different sequences of a fixed length of *k* nucleotides (*k*-*mers*) and were defined as single-*k*-*mer* (SK) and multi-*k*-*mer* (MK). In the SK method, the pre-assembly was generated using a de Bruijn graph based on the de novo transcriptome Trinity Assembler v2.0.4 [20], with a default *k*-*mer* parameter (*K* = 25). In parallel, a MK approach using Trans-ABySS v1.5.1 [21] and rnaSPAdes v0.1.1 (available at http://bioinf.spbau.ru/en/rnaspades) [22] was carried out. In the pre-assembly step with Trans-ABySS, we used *k*-*mer* values of *K* = 17, 21, and 25. The three raw transcriptomes assembled with different *k*-*mers* were then merged using the ‘transabyss-merge’ option. Whereas the pre-assembly step performed with rnaSPAdes used two *k*-*mer* values (*k* = 21 and 33). For both SK and MK methods, the minimum transcript length was set to 200 bp. The different assemblies were performed using a 16-core Intel^®^ Xeon(R) CPU E5-2650 v2 workstation with 64 GB of RAM.

In the second step of the assembly procedure, the pre-assemblies from SK and MK approaches were clustered using CD-HIT v4.6.4 [23]. The identity and the word size settings were 0.95 and 10, respectively, merging clusters with an alignment overlap above 95% identity to generate an overall leaf transcriptome assembly. The processed sequences were further subjected to the EvidentialGene tr2aacds pipeline (available at http://arthropods.eugenes.org/EvidentialGene/about/EvidentialGene_trassembly_pipe.html) [24] to generate a final assembly containing an NR unique sequence (unitranscript) dataset for *A. donax* leaf transcriptome. The tr2aacds pipeline aims to select an ‘optimal’ set of de novo assembled transcripts, based on coding potential, from a pool of RNA sequences. First, the algorithm generates coding sequences (CDS) and amino acid sequences for each assembled transcript, and then removes redundant sequences using the amino acid information in order to select the best coding sequences among identical sequences. The pipeline also implements self-on-self searches to identify highly similar sequences using the Basic Local Alignment Search Tool (BLAST). The alignment results and CDS/protein identities are subsequently used to select and output transcripts organized as primary or alternate. The transcripts classified as dropped did not pass the internal filters and were rejected. The primary and alternate transcripts were used for further assessments.

### Reads mapping back to transcriptome (RMBT)

Two aligners were used for the mapping of reads back to the leaf transcriptome catalogue: Bowtie2 v2.1.0 [25] and Burrows–Wheeler Alignment (BWA) tool v0.7.12 [26]. The ‘end-to-end’ and the ‘sensitive’ options were used for the alignment using Bowtie2, in which the max number of mismatches (-N = 1) was allowed in the seed alignment. For BWA, the ‘bwtsw’ algorithm was used to index the leaf transcriptome database, and the ‘aln’ (with -n = 0.01; i.e., for 51-bp reads and max_diff 4) and ‘samse’ options to align reads. SAMtools v0.1.19-96b5f2294a [27] was used to calculate mapping statistics from BAM files.

### Full-length transcript analysis

The de novo leaf transcriptome assembly was compared to five databases from the Ensembl Plants FTP server (release-29, ftp://ftp.ensemblgenomes.org/pub/plants/release-29/) [28]: barley (*Hordeum vulgare*) coding sequence v1.29 (August 2014), rice (*Oryza sativa*) coding sequence IRGSPv1.0.29, sorghum (*Sorghum bicolor*) coding sequence v1.29, common wheat (*Triticum aestivum)* coding sequence IWGSC1.0 + popseq.29, and foxtail millet (*Setaria italica*) coding sequence JGIv2.0.29, using NCBI-BLAST v2.2.28+. BLASTn searches were performed with the following parameters: -task dc-megablst, -evalue 1e−20, -max_target_seqs 1, and -outfmt 6. In addition, the ‘analyze_blastPlus_topHit_coverage’ script from the Trinity software package (available at https://github.com/trinityrnaseq/trinityrnaseq/wiki) [29] was used to calculate the target database alignment coverage and to assess the number of queries that were assembled to full-length and specific length thresholds, respectively, in the de novo assembly.

### Assessment of the completeness of *A. donax* leaf transcripts

The quality and completeness of the de novo assembly were further evaluated using BUSCO software v2.0 [30]. This quality assessment tool provides high-resolution quantifications for genomes, gene sets, and transcriptomes and checks whether each of the BUSCO group is complete, duplicated, fragmented, or missing in the genome or transcriptome assembly. The leaf unitranscripts were compared to the set of Embryophyta genes, which contains 1440 BUSCO groups from a total of 31 species in order to obtain a quantitative measure of the transcriptome completeness, based on evolutionarily informed expectations of gene content from near-universal single-copy orthologs [30].

### Functional annotation of the leaf transcriptome catalogue

TransDecoder v2.0.1 (available at http://transdecoder.github.io) [31] was used to identify open reading frames (ORFs) and to predict potential coding sequences using the assembled unitranscripts as input. After ORFs were extracted from the assembly, the Trinotate v2.0.2 (available at http://trinotate.github.io) [32] pipeline was used to annotate the leaf transcript ORF dataset. Both nucleotide transcripts and protein sequences were used to search against the UniProtKB/Swiss-Prot (uniprot_sprot.trinotate_v2.0.pep.gz) and UniRef90 (uniprot_uniref90.trinotate_v2.0.pep.gz) databases (downloaded at https://data.broadinstitute.org/Trinity/Trinotate_v2.0_RESOURCES/) [33], using NCBI-BLASTx and BLASTp v2.2.28+ (-evalue 1e−10 -max_target_seqs 1 -outfmt 6), respectively. The UniProtKB is a collection of functional information on proteins, with accurate, consistent, and rich annotation; the section Swiss-Prot contains manually annotated records. The UniRef databases provide NR clustered sets of sequences from the UniProtKB (including isoforms) and the UniProt Archive (UniParc) records (a comprehensive and NR database that contains most of the publicly available protein sequences). Functional domains were identified using the Pfam domain database (Pfam-A.hmm.gz, available at https://data.broadinstitute.org/Trinity/Trinotate_v2.0_RESOURCES/) [33] using HMMER v3.1b2 [34]. Potential signal peptides were identified using SignalP v4.1 [35]. All the leaf transcriptome annotations were loaded into the SQLite database (Trinotate.sprot_uniref90.20150131.boilerplate.sqlite.gz, downloaded at https://data.broadinstitute.org/Trinity/Trinotate_v2.0_RESOURCES/) [33]. The maximum e-value for reporting the best hit and associated annotation was 1e−5.

Blast2GO v3.1 [36] was used to retrieve the leaf unitranscripts related to GO and to reveal biological pathways. Briefly, the assembled transcripts were aligned using the CloudBlast [37] service targeted against the NCBI NR protein database with default parameters. The matching transcripts were further processed to detect associated GO terms, describing biological processes (BPs), molecular functions (MFs), and cellular components (CCs). UniProtKB, Gramene Proteins Database (GR_protein), Protein Data Bank (PDB), and The *Arabidopsis* Information Resource (TAIR) mapping databases were used to identify GO categories. The InterProScan (IPS) function in the Blast2GO software was further used to retrieve protein domains and motif information. Blast2GO also produces the enzyme code (EC) numbers for transcripts with an e-value less than 1e−5. Subsequently, transcripts with EC numbers were identified and mapped to the KEGG database.

We further created custom databases of *A. thaliana* and *S. italica* genes involved in the biosynthesis of particular cell wall components (lignin and cellulose), which should aid in the optimization of biofuel production from lignocellulosic biomass, the development of bioenergy crops, and in improving drought tolerance through stomatal development and distribution. We also created a database for SAP genes, which are potential candidates for biotechnological approaches aiming to improve abiotic stress tolerance in plants. BLASTn searches were performed against the custom databases with an e-value cut-off of 1e−5.

### Identification and distribution of functional genic-SSRs and development of polymorphic SSR markers

The *A. donax* leaf unitranscripts were scanned for single sequence repeat (SSR) markers using the MISA (MIcroSAtellite identification tool) program downloaded from the Leibniz Institute of Plant Genetics and Crop Plant Research website (http://pgrc.ipk-gatersleben.de/misa/) [38]. Only perfect SSRs including mono-, di-, tri-, tetra-, penta-, and hexa-nucleotide motifs with numbers of uninterrupted repeat units more than 10, 6, 5, 5, and 5, respectively, were targeted. The maximum interruption distance between two SSRs in a compound microsatellite was set to 100 bases. Furthermore, the distribution of the SSR classes within *A. donax* leaf unitranscripts with respect to the untranslated (5′-UTR and 3′-UTR) and the coding sequence (CDS) regions in the identified ORFs was investigated. First, TransDecoder v3.0.0 (available at http://transdecoder.github.io) [TransDecoder] with the ‘single best orf’ option was used to retrieve the best 5′-UTR, CDS, and 3′-UTR regions. Then, the positions of the transcript domains were combined with the SSRs’ positions to assign microsatellite location within the different ORF regions.

In order to retrieve candidate polymorphic SSRs (PolySSRs) among EcoA, EcoB, and EcoC, the leaf transcriptome of each ecotype was de novo assembled using Trinity software v2.0.4 [20] with the default parameters (*K* = 25 and a minimum transcript length of 200 bp). Clustering of redundant transcripts was performed with 95% identity and a word size of 10 using CD-HIT v4.6.4 [23]. The CandiSSR pipeline (available at http://www.plantkingdomgdb.com/CandiSSR/index.html) [39] was used to identify microsatellite polymorphisms between *A. donax* unitranscripts and the ecotype-specific leaf transcriptome assemblies. This procedure (i) employs MISA software to identify repeat sequences in the reference transcriptome (using the criteria for SSR identification described above); (ii) retrieves the flanking regions (100 bp both upstream and downstream) of the identified SSRs in the reference sequences; (iii) aligns them using BLAST to the non-reference transcriptome (e.g., among different individuals); (iv) filters out the low-quality hits; (v) extracts the non-reference sequences with the flanking regions; (vi) searches for the specific reference SSRs within them; (vii) removes the invalid search items; (viii) filters out the low-quality PolySSRs; (ix) calculates the sequence similarity of the flanking regions of the identified PolySSRs; and finally (x) designs primer pairs for each PolySSR using Primer3 (http://primer3.sourceforge.net/) [40, 41], assessing also the global similarity of the primer binding regions. Parameters for the primer design were as follows: minimum, maximum, and optimal sizes were 18, 24, and 20 nt; minimum and maximum GC contents were 40 and 60%; and minimum and maximum *T*_m_ values were 52 and 63 °C, respectively.

Blast2GO v3.1 [36] was used to functionally annotate the transcripts containing the PolySSRs, with the aim to analyze the GO categories and KEGG metabolic pathways of these unitranscripts.

### Validation of PolySSR markers by PCR and Sanger sequencing

In order to validate microsatellites identified in the *A. donax* leaf transcriptome, a total of five primer pairs were randomly selected from those obtained from the CandiSSR pipeline (i.e., forward and reverse primer pairs n.1 of CPSSR_1|AAG, CPSSR_3|AGA, CPSSR_4|AGC, CPSSR_5|AGC, and CPSSR_9|CAG; see Additional file 9: Table S9) to be synthesized (Eurofins Genomics, Milan, Italy) and used for screening polymorphisms among the three ecotypes. Genomic DNA was extracted using the ‘DNeasy Plant Mini kit’ (Qiagen, Hilden, Germany) according to the manufacturer’s instructions. Polymerase chain reactions (PCRs) were conducted in a total volume of 25 μl containing 5 μl of 5× colorless GoTaq^®^ reaction buffer, 0.80 μl of 25 mM MgCl_2_, 0.50 μl of 10 mM dNTP mix, 0.10 μl of 5 μ μl^−1^ GoTaq^®^ DNA polymerase (Promega™, Madison, USA), 0.65 μl of 10 μM (0.25 μM) sense and antisense primers, and 20 ng of template DNA. The PCR mixture was subjected to 94 °C for 3 min, followed by 35 cycles of 45 s at 94 °C, annealing for 30 s at 55–60 °C (based on the melting temperature of primers), 25 s at 72 °C, and a final step at 72 °C for 2 min. PCR products were visualized via 3% MetaPhor gel and ethidium bromide staining and purified using ExoSAP-IT^®^ treatment (Amersham Biosciences, Buckinghamshire, UK), according to the manufacturer’s instructions. Purified PCR products were sequenced on an ABI 3730XL at Eurofins Genomics (Milan, Italy). Finally, the presence of the polymorphic SSR fragment between the three *A. donax* ecotypes was assessed using the sequence alignment editor BioEdit v7.2.5 (http://www.mbio.ncsu.edu/bioedit/bioedit.html) [42].

## Results

### Sequencing of the *A. donax* leaf transcriptome

**Fig. 1:**
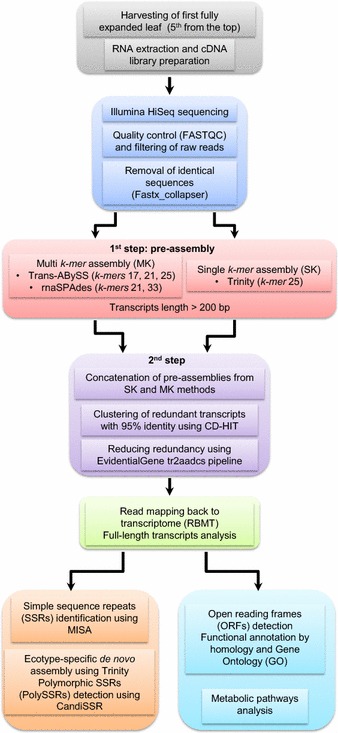
Flowchart of the pipeline for the *A. donax* leaf transcriptome sequencing, de novo assembly, annotation, and analysis. The pipeline performs multiple operations from sampling and preparation to sequencing and de novo assembly to functional annotation and analysis. First, mRNA extraction from the first fully expanded leaf (5th from the *top*) was carried out followed by cDNA preparation and library construction (*gray*). Sequencing was performed using an Illumina HiSeq platform. The sequenced reads were then subjected to quality control and filtering, and identical sequences were removed (*blue*). Next, de novo assemblies of transcripts were generated using a two-step approach: first, multi-*k*-*mer* (Trans-ABySS and rnaSPAdes) and single-*k*-*mer* (Trinity) methods were used to generate the pre-assemblies (*pink*); second, pre-assemblies were then concatenated and redundant transcripts were removed using CD-HIT and the EvidentialGene tr2aadcs pipeline (*purple*). The quality of the de novo assembled leaf transcriptome was then assessed (*green*). Finally, the non-redundant (NR) transcript dataset was functionally annotated by homology and gene ontology (GO), and metabolic pathways were analyzed (*mint blue*). Simple sequence repeats (SSRs) and polymorphic SSRs (PolySSRs) were also identified (*orange*)

A flowchart overview of the steps followed in the assembly process is outlined in Fig. 1. To obtain a broad sample and accurate estimate of the *A. donax* leaf transcriptome, 54 independent cDNA libraries were constructed from leaves sampled from three biological replicates of three ecotypes at three time points, and under two water stress treatments. All 54 samples were subjected to Illumina RNA-Seq. A total number of 1252 million 50-bp single-end (SE) reads from the 54 fastq files accounting for 187.1 Gb of raw data were generated from the Illumina sequencing with a GC content ranging from approximately 47 to 50%. Prior to the de novo assembly step, raw reads were analyzed to assess quality metrics using the FASTQC software. All files contained high-quality reads with Phred scores ranging from 33 to 37 (Phred quality score, Q, is a measure of base-calling quality and is inversely related to the probability that the corresponding base call is incorrect; a Q30 threshold is considered of high quality). We excluded 2.85 million reads containing uncalled bases (N) using inhouse-developed scripts in Python. We further collapsed identical sequences into a single sequence using Fastx_collapser and then concatenated the resulting files into a unique file that we used for the subsequent assembly step (Fig. 1). This provided a dataset of 1249 million of high-quality unique reads representing the leaf transcriptome at three different developmental stages of three different ecotypes grown in two contrasting conditions and as such encompasses a large proportion of the leaf transcriptome in the species *A. donax*.

### Leaf transcriptome de novo assembly of *A. donax*

Since no genome-wide DNA sequence is available for *A. donax* for alignment purposes, we developed a specialized bioinformatics workflow for de novo transcriptome assembly (Fig. 1). Accurate reconstruction and distinction between highly similar transcripts of homologous and paralogous genes as well as transcript isoforms in polyploid species present substantial challenges for de novo transcriptome assembly. For this reason, to optimize transcriptome assembly and obtain a high-quality transcriptome, it is useful to combine the output of different de novo assemblers using a range of *k*-*mer* sizes, while minimizing sequence redundancy [43]. Hence, the pre-assembly step was performed using two parallel approaches: a SK method using Trinity software and a MK method using Trans-ABySS and rnaSPAdes. The Trinity pre-assembly generated 136,294 transcripts with a total length of 74,660,373 bp. The median length of these transcripts (N50 value) was 659 bp and the mean and maximum transcript lengths were 547 and 10,795 bp, respectively (Table 1). N50 is defined as the length N for which 50% of all bases in the assembly are in a contig of length smaller than N [44]. The MK approach using rnaSPAdes resulted in 138,896 raw transcripts with a total length of 66,613,178 bp, a mean and a maximum transcript length of 479 bp and 8173 bp, respectively. The N50 value was 538 bp (Table 1). Finally, the MK approach using Trans-ABySS produced 158,095 transcript sequences with a total length of 66,613,178 bp, a mean and a maximum transcript length of 465 bp and 10,360 bp, respectively. The N50 value was 520 bp (Table 1).

**Table 1:** Statistics of the leaf transcriptome pre-assemblies and the final de novo assembly of *A. donax*

Metric	Assembler			Final transcriptome
	Trinity^a^	rnaSPAdes^b^	Trans-ABySS^c^	
Number of sequences	136,294	138,896	158,095	62,596
Total nucleotide count (bp)	74,660,373	66,613,178	73,637,461	52,719,740
Max. transcript length (bp)	10,795	8173	10,360	10,360
Mean transcript length (bp)	547	479	465	842
N50 (bp)	659	538	520	1134

Summary statistics of the *A. donax* pre-assemblies using Trinity, rnaSPAdes, and Trans-AbySS, and of the final *A. donax* leaf transcriptome after clustering and redundancy removal

^a^Trinity: *K*-*mer* 25

^b^rnaSPAdes: *K*-*mer* 21, 33

^c^Trans-ABySS: *K*-*mer* 17, 21, 25; the minimum transcript length was set to 200 bp

To reduce redundancy and possible artifacts due to the ploidy level of *A. donax*, the pre-assemblies from the SK and MK methods were subsequently merged and clustered using the CD-HIT program. The processed sequences were further subjected to the EvidentialGene tr2aacds pipeline in order to select the best biological dataset of primary and alternative transcripts. The resulting final leaf unitranscript catalogue consisted of 62,596 NR unique sequences with a considerably increased N50 value of 1134 bp and a mean transcript length of 842 bp, covering a total nucleotide count of 52,719,740 bp with an average GC content of 49% (Table 1).

**Fig. 2:**
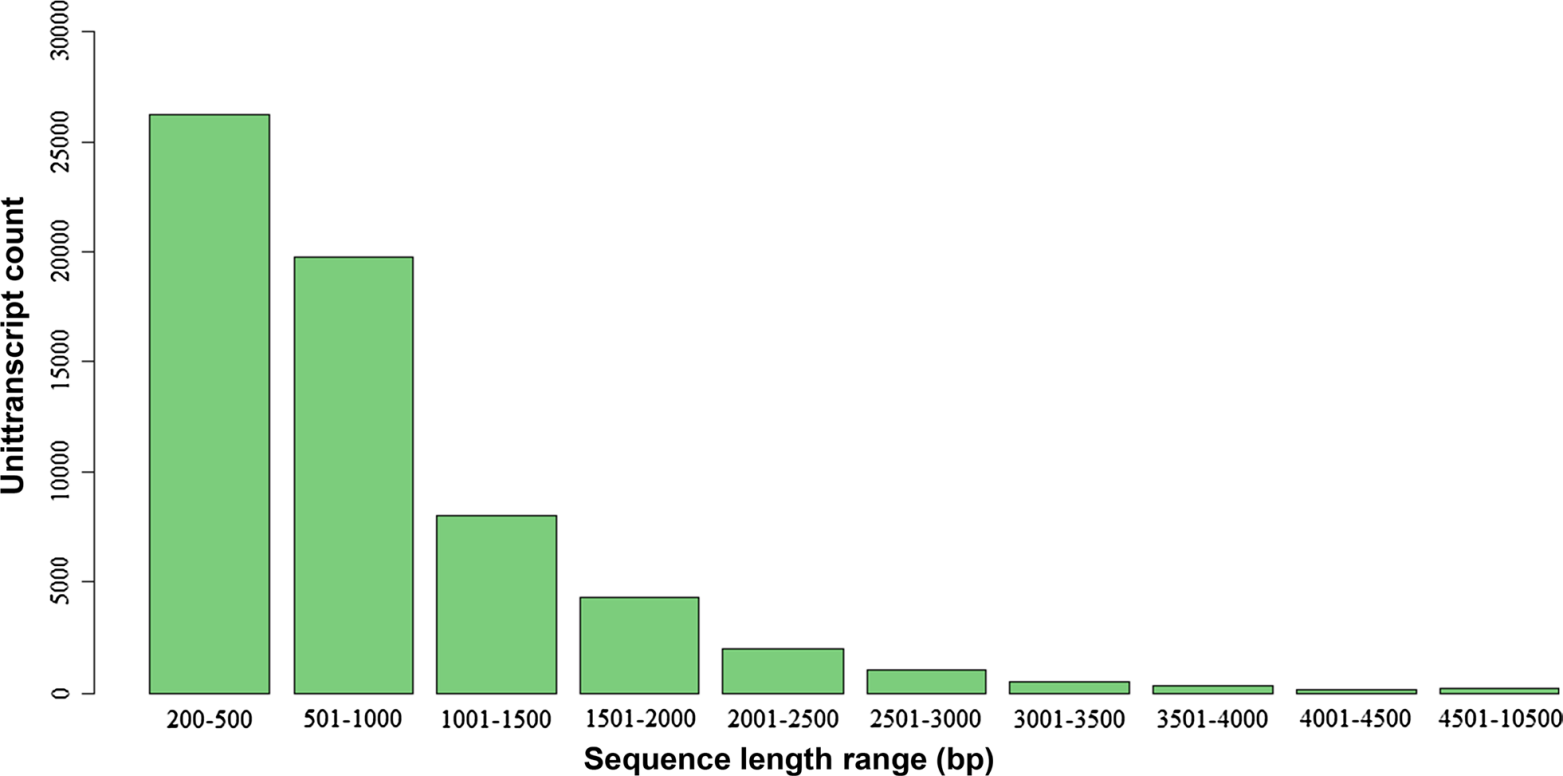
Sequencing and de novo assembly of *A. donax* leaf transcriptome. Sequence length distribution of *A. donax* non-redundant (NR) unique unitranscript sequences. The *X-axis* represents the length range bins in bp. The *Y-axis* represents the frequency of transcripts in each bin

The assembled sequence lengths ranged from the 200 bp cut-off value to a maximum transcript length of 10,360 bp. The majority of the assembled sequences were in the ranges of 200–500 and 501–1000 bp, while the frequency of longer transcripts gradually decreased and only a minor proportion reached lengths above 3000 bp (Fig. 2).

### Quality assessment of the leaf transcriptome assembly

To evaluate the assembly consistency, the filtered unique reads were mapped back to the final assembled leaf transcriptome, taking into account the number of mismatches when mapping reads to the assembled transcripts to tolerate the average differences due to the potential genome divergences, as previously reported in wheat [45, 46]. The overall alignment rates using the alignment software Bowtie2 and BWA were 74.27 and 68.35%, respectively. About 40% of the reads uniquely mapped to a single assembled transcript using either aligner, whereas 34.18 and 25.82% of the reads aligned to more than one transcript (Table 2).

**Table 2:** Percentage of reads mapped back to *A. donax* leaf transcriptome

	Aligner	
	Bowtie2	BWA
Reads aligned 1 time (%)	40.09	42.53.38
Reads aligned >1 times (%)	34.18	25.82
Overall alignment rate (%)	74.27	68.35

Percentage of reads uniquely mapped, aligned more than one time to the unitranscripts, and overall alignment rate using Bowtie2 and BWA aligners

We further assessed the level of homology between our leaf transcriptome dataset and those from related species of the Poaceae family in order to evaluate how many transcripts were reconstructed at full length and those partially reconstructed. A considerable proportion of the *A. donax* sequences aligned completely (100% of coverage) or virtually (70% of coverage) to the transcripts of related species. The highest number of hits was retrieved when *A. donax* sequences were aligned to the *H. vulgare* and *T. aestivum* transcriptomes. However, only a slightly smaller number of hits were detected for alignment against the *S. bicolor, O. sativa*, and *S. italica* transcriptomes (Table 3).

**Table 3:** Full-length transcripts’ analysis

Template transcript dataset	100% coverage	>70% coverage	>20% coverage
*H. vulgare*	6526	11,443	17,430
*T. aestivum*	6503	12,385	21,661
*S. bicolor*	6281	10,851	16,521
*O. sativa*	6176	11,283	16,631
*S. italica*	6066	11,039	17,378

Percentage *A. donax* leaf transcripts aligned completely (100%), virtually (>70%), or partially (>20%) to the transcripts of related species

We also checked the quality of our assembled unitranscripts by comparing them to the set of Embryophyta genes using BUSCO quality assessment tool [30]. Out of the 1440 BUSCO groups searched, 70.07% (1009 BUSCOs) were ‘complete’ (i.e., 813 single-copy and 196 duplicated), 13.19% (190 BUSCOs) were ‘fragmented,’ and the remaining 16.74% (241 BUSCOs) were ‘missing.’

### Functional annotation of the leaf transcriptome by homology and GO

To determine putative functions of the identified *A. donax* unitranscripts, we adopted the Trinotate pipeline to detect homology between the predicted *A. donax* ORFs/potential proteins and sequences deposited in universal databases. ORFs and potential coding sequences were first predicted using the TransDecoder software. From the initial leaf unitranscript catalogue of 62,596 NR unique sequences, 98,781 ORFs and 83,758 potential proteins could be predicted. The retrieved nucleotide sequences and putative protein sequences were then functionally annotated using the Trinotate pipeline, searching for nucleotide (BLASTx) and protein (BLASTp) homology (e-value <1e−10) against the UniProtKB/Swiss-Prot and UniRef90 databases. In addition, the presence of known functional protein domains and potential signal peptides was analyzed using the Pfam protein domain database and SignalP, respectively. In total, 34,177 nucleotide sequences (54.59%) and 33,597 protein sequences (34.01%) displayed significant homology when aligned against the UniProtKB/Swiss-Prot database using BLASTx and BLASTp searches, respectively. When aligned against the UniRef90 database, the number of homologous nucleotide and protein sequences increased to 50,850 (81.23%) and 54,660 (55.33%), respectively. Furthermore, 31,513 (31.90%) unique Pfam protein motifs could be assigned and 2729 (2.76%) protein sequences were predicted to have signal peptides (Table 4). Among the Pfam domains, the most abundant classes were leucine-rich repeat (LRR), pentatricopeptide repeat (PPR), and AAA domains. These protein domains can be found in proteins involved in various functions such as protein–protein interactions, transcription regulation, signal transduction, and protein degradation. The functional annotation by homology is available in LabArchives at doi:10.6070/H40V89V1).

**Table 4:** Overview of functional annotation by homology

Category	No. of transcripts
Predicted ORFs	98,781
Predicted proteins	83,758
Tr_EMBL_Top_BLASTX_hit	50,850
sprot_Top_BLASTX_hit	34,177
Tr_EMBL_Top_BLASTP_hit	54,660
sprot_Top_BLASTP_hit	33,597
Pfam	31,513
SignalP	2729

Summary of the functional annotation by homology

ORFs, open reading frames; Tr_EMBL_Top_BLASTX_hit, top blastx hits against UniRef90 database; sprot_Top_BLASTX_hit, top blastx hits against UniProtKB/Swiss-Prot database; Tr_EMBL_Top_BLASTP_hit, top blastp hits against UniRef90 database; sprot_Top_BLASTP_hit, top blastp hits against UniProtKB/Swiss-Prot database; Gene_ontology_blast, gene ontology using Blast; Gene_ontology_pfam, gene ontology using Pfam

**Fig. 3:**
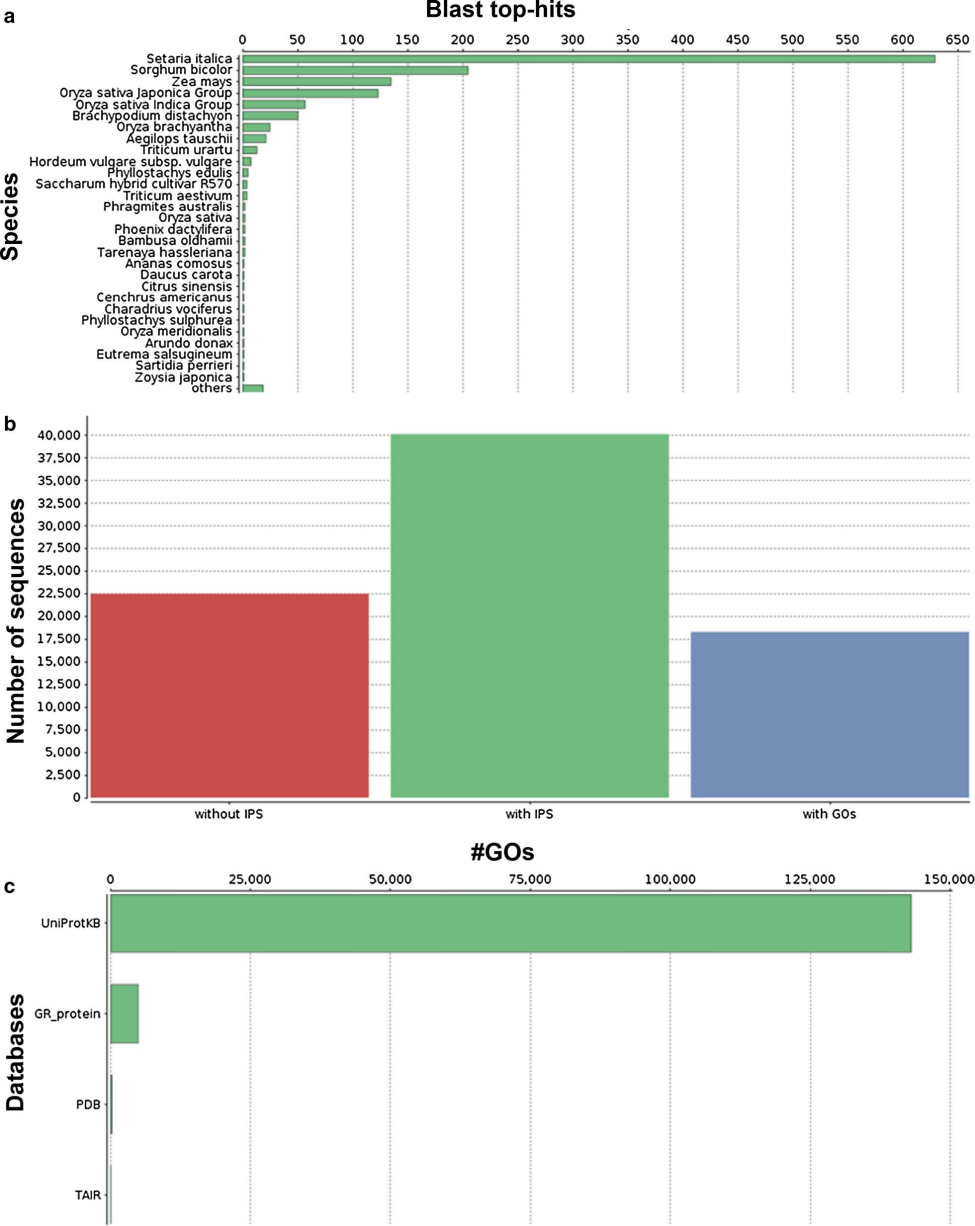
Graphical representations of functional annotations in *A. donax* leaf transcriptome. **a** BLAST top-hits species distribution of *A. donax* unitranscripts against the non-redundant (NR) protein database. **b** Histogram of leaf transcriptome sequences with InterPro domains and gene ontology (GO) terms. The *X-axis* represents transcripts without InterProScan (IPS), with IPS, and with GO. The *Y-axis* shows the frequency of transcripts in each bin. **c** Representation of mapping database (UniprotKB, GR_protein, PDB, TAIR) sources

**Fig. 4:**
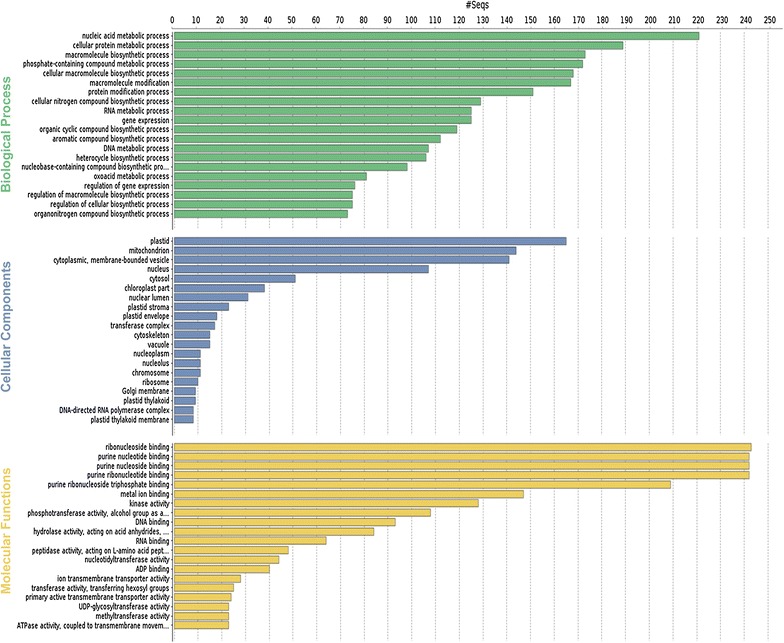
Gene ontology (GO) functional classification using Blast2GO. Histograms of the frequency of transcripts annotated to specific GO categories; biological process, cellular components, and molecular functions are represented by *green*, *blue*, and *yellow bars*, respectively

In addition, to further functionally characterize the *A. donax* leaf transcriptome we related the detected transcripts to GO and biological pathways using the Blast2GO software. First, we assessed the blast hit distribution of the 62,596 unitranscripts blasted against the NCBI NR protein database. Our transcript dataset displayed 26,323 (42.05%) hits with the NR database and the highest sequence similarity (the lowest e-value) to *S. italica* (629 top-hits), followed by *S. bicolor* (205 top-hits), *Zea mays* (135 top-hits), and *O. sativa* ssp*. japonica* (123 top-hits) (Fig. 3a). We further assessed the number of transcripts with InterPro (IP) sequence motifs and associated GO terms. About 64% (40,116) of *A. donax* unitranscripts contained IP motifs, of which about 46% (18,293) were associated to GOs terms (Fig. 3b). The mapping databases used in the Blast2GO suite were UniProtKB, GR_protein, PDB, and TAIR (Fig. 3c). GO analysis revealed 9778 sequences associated to 820 GO terms. Among the three main categories, BP category was the most abundant (6299 sequences, 520 GOs), followed by MF (2441 sequences, 151 GOs) and CC (1038 sequences, 104 GOs) categories (Additional file 3: Table S1). Within the BP category, metabolic processes (28.75%), biosynthetic processes (25.37%), and cellular processes (17.38%) were most represented. Likewise, genes encoding binding proteins (24.66%) and genes encoding proteins related to catalytic activities (22.45%) were most abundant in the MF category. In the CC category, cell (63.20%), cell part (63%), organelle (54.14%), and membrane (37.18%) were the most abundantly represented GO terms (Fig. 4). These numbers indicate the importance of essential metabolic and biosynthetic processes in *A. donax*. However, a large proportion of transcripts showed no similarity to any known putative protein in the public databases, particularly in NCBI NR protein and UniProtKB/Swiss-Prot BLAST searches.

### Biological pathway analyses in *A. donax*

In order to investigate functional biological pathways in *A. donax*, we exploited the Blast2GO function that assigns EC numbers to transcripts with a blast hit e-value ≤1e−5. The largest proportion of such retrieved transcripts encoded for proteins with EC numbers related to transferase activity (33.53%), followed by hydrolases (29.48%) and oxidoreductases (13.30%). Next, transcripts with assigned EC numbers were mapped against the KEGG database to identify the putative presence of curated pathway components in *A. donax*. Among the 128 KEGG pathways analyzed (Additional file 4: Table S2), nucleotide metabolism, specifically purine metabolism (929 sequences), was the most represented pathway in terms of the number of homologous leaf transcripts. Furthermore, thiamine metabolism (749 sequences), metabolism of terpenoids and polyketides and other secondary metabolites (370 sequences), aminobenzoate degradation (252 sequences), and starch and sucrose metabolism (240 sequences) were highly represented.

**Fig. 5:**
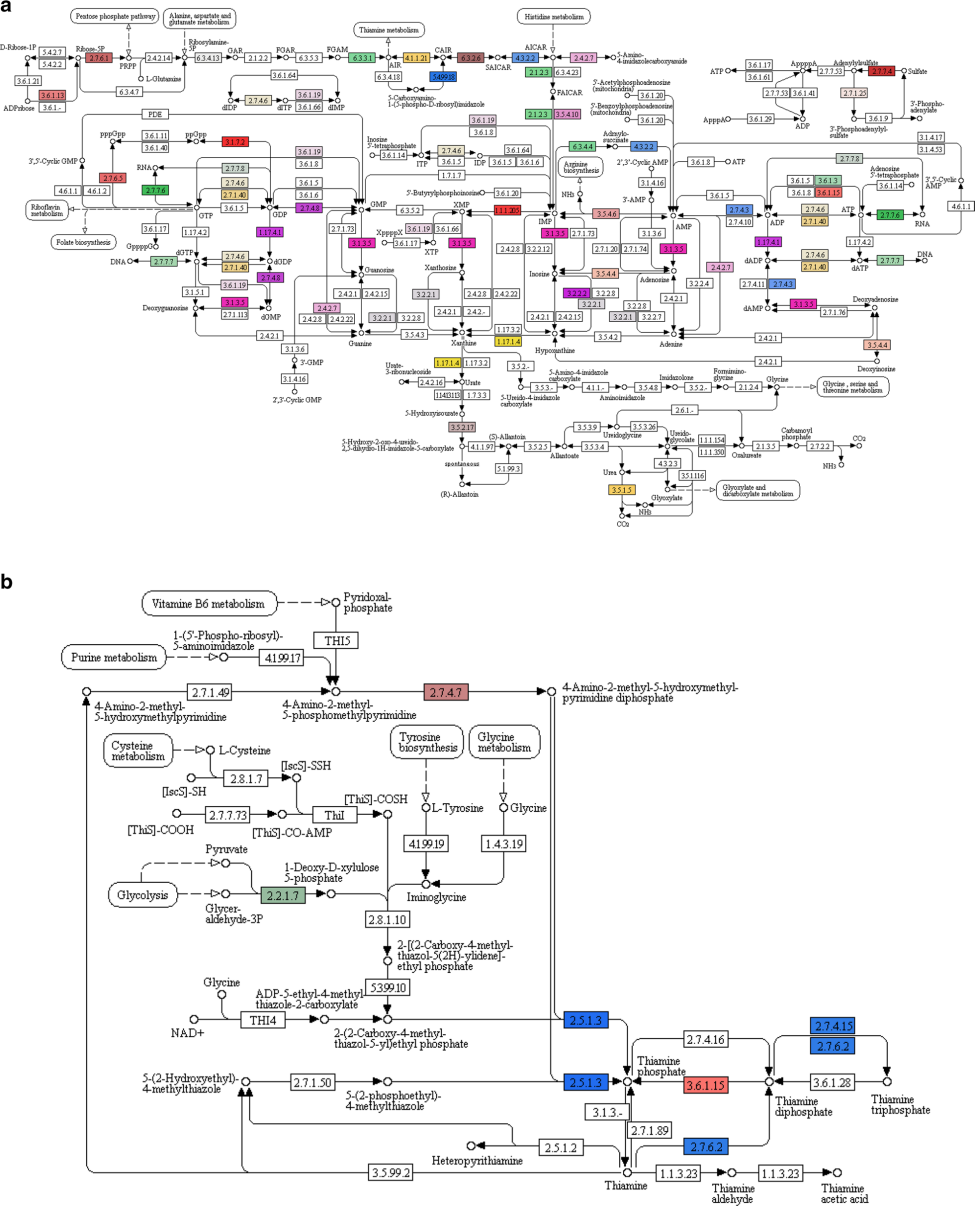
Study of purine metabolism (**a**) and thiamine metabolism (**b**) pathways by Kyoto Encyclopedia of Genes and Genomes (KEGG) analysis showing the different identified enzymes in *A. donax* leaf transcriptome (*one color* for each Enzyme Code or EC)

Because of its high representation and the putative role of adenine in triggering abiotic stress tolerance, leading to increased plant biomass [47], we further analyzed purine metabolism. In the *A. donax* leaf transcriptome, 926 homologs to 36 purine metabolism genes were detected, suggesting high numbers of paralogs, possibly due to the high level of polyploidization into *A. donax*. Particularly, proteins with nucleosidase activity involved in adenine, hypoxanthine, xanthine, and guanine biosynthesis (EC 3.2.2.1), proteins with xanthine dehydrogenase activity (EC 1.17.1.4), and the enzyme involved in the first stage of conversion of 5-hydroxyisourate to S-allantoin (EC 3.5.2.17) were highly abundant in the transcripts catalogue. The *A. donax* leaf transcriptome dataset further included several additional enzymes involved in the biosynthesis of various compounds of purine metabolism, allowing the reconstructing of large segments of the pathway (Fig. 5a).

The second most represented pathway encompassed the metabolism of thiamine, a universal cofactor involved in common metabolic pathways, including biotic and abiotic stress responses in plants. In the *A. donax* leaf transcript dataset, 749 sequences homologous to 5 thiamine metabolism genes were identified, highlighting the importance of this pathway in *A. donax*. Particularly, enzymes involved in the production of thiamine phosphates, such as thiamine diphosphokinase (EC 2.7.6.2) converting thiamine in thiamine diphosphate, and thiamine phosphatase (EC 3.6.1.15) converting thiamine diphosphate in thiamine phosphate, were retrieved (Fig. 5b).

**Fig. 6:**
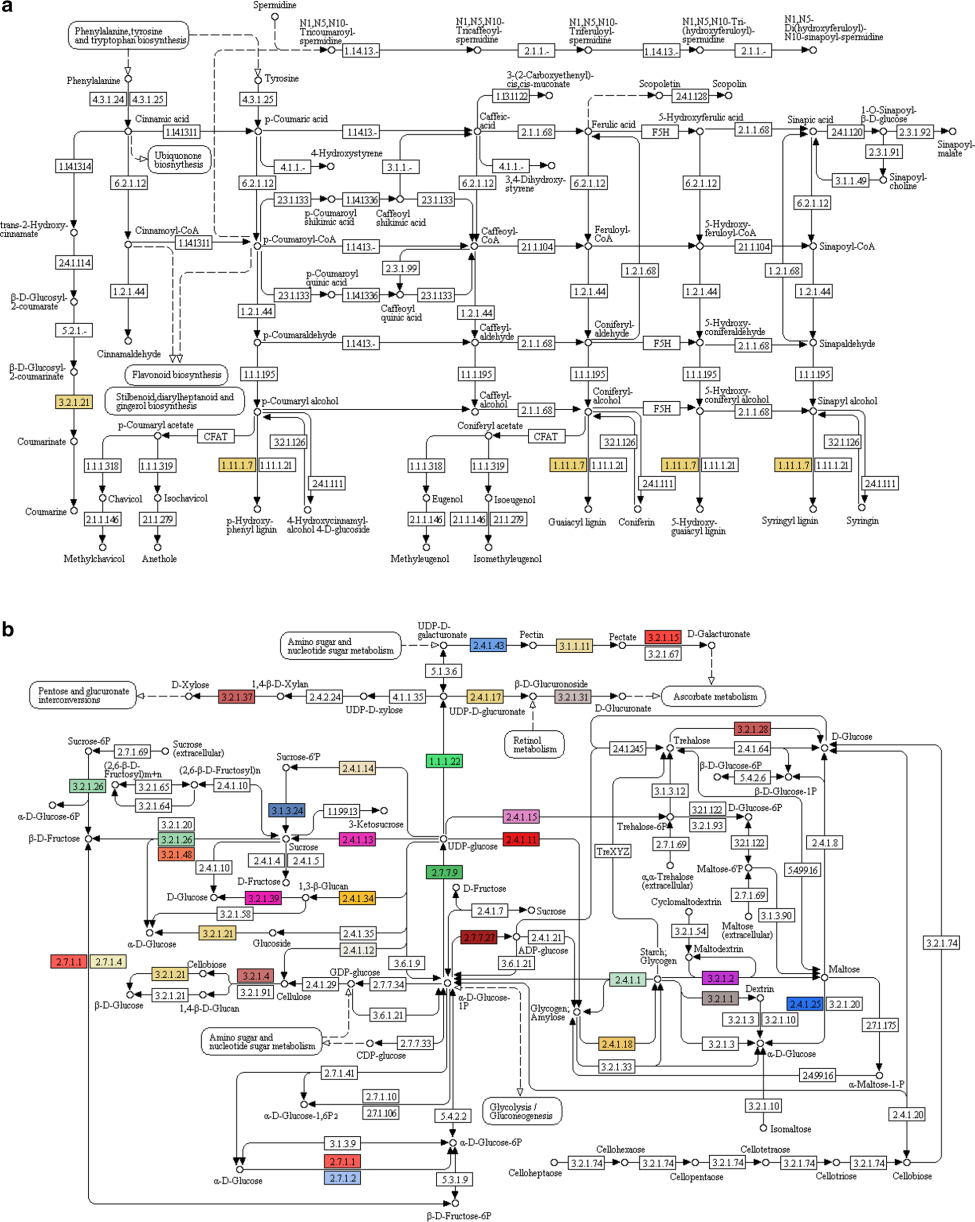
Kyoto Encyclopedia of Genes and Genomes (KEGG) analysis showing genes involved in phenylpropanoid biosynthesis (**a**) and starch and sucrose biosynthesis (**b**) representing each colored EC in *A. donax* leaf transcriptome

Considering the substantial economic relevance of lignin metabolism in bioenergy crops, the *A. donax* leaf unitranscripts were also analyzed for coding sequences of the phenylpropanoid biosynthesis pathway. Although this pathway was not among the most represented ones, 44 *A. donax* transcripts mapped to a peroxidase (EC 1.11.1.7), which is involved in the last step of guaiacyl, hydroxyl-guaiacyl, syringyl, and hydroxyl-phenyl lignin biosynthesis, and also to a beta-glucosidase (EC 3.2.1.21), which is involved in the synthesis of coumarinate (Fig. 6a). This indicates that *A. donax* is capable of synthesizing various types of lignin, provided that precursors are produced as well. However, despite its importance, lignin is not considered as the only target for improving the yield and quality of lignocellulosic biomass in bioenergy crops. A pivotal role toward this goal is assigned to polysaccharides. Therefore, the presence of transcripts coding for starch and sucrose metabolism was also verified in the *A. donax* leaf transcripts catalogue, identifying 30 different enzymes involved in this pathway (Fig. 6b). Among others, cellulose synthase (EC 2.4.1.12) involved in the synthesis of cellulose from UDP glucose and cellulase (EC 3.2.1.4) involved in cellulose catabolism were identified. In addition, sucrose-phosphate synthase (EC 2.4.1.14) responsible for the phosphorylation of sucrose into sucrose-6-phosphate, could also be retrieved. Moreover, the *A. donax* leaf transcriptome contained several enzymes involved in the biosynthesis of amylopectin and glycogen, such as the branching enzyme (EC 2.4.1.18) (Fig. 6b). The identification of these crucial genes in biomass production might provide important targets for bioengineering approaches.

To complete the analysis of carbohydrate metabolism in *A. donax*, the occurrence of components of the carbon fixation pathway in the leaf transcriptome was also explored and 15 different enzymes could be retrieved (Additional file 5: Figure S2). A large proportion of the carbon fixation pathway could thus be reconstructed, including, among others, phosphoglycerate kinase (EC 2.7.2.3) involved in the synthesis of 1,3-bisphopsho-glycerate, and aldolase (EC 4.1.2.13) required for the conversion of glyceraldehyde-3-phosphate into fructose-1,6-diphosphate and of erythrose-4-phosphate into sedoheptulose-1,7- bisphosphate. Furthermore, the phosphoenolpyruvate carboxylase enzyme (EC 4.1.1.31) could be identified in the leaf transcripts. Finally, the malate dehydrogenase enzyme (EC 1.1.1.37) involved in malate biosynthesis and pyruvic-malic carboxylase (EC 1.1.1.39) involved in the conversion of malate into pyruvate were detected in the *A. donax* transcriptome (Additional file 5: Figure S2).

### Interspecies comparisons of *A. donax* pathways

Since the KEGG pathways accumulate enzymes and possible conversions of many species, it is difficult to estimate how this complexity relates to the presence of pathway components in single species. We, therefore, analyzed sequence similarity of the *A. donax* transcripts to its most related species *S. italica*, *O. sativa*, *S. bicolor*, and the plant model species *A. thaliana*. Transcripts involved in the biosynthesis of certain cell wall compounds (cellulose and lignin), in stomatal development, and transcripts encoding for SAPs homologous to those species were identified in the *A. donax* leaf transcriptome.

The *cellulose synthase* superfamily encompasses thirteen *cellulose synthase A* (*CesA*) genes and thirty-six *cellulose synthase*-*like* (*Csl*) genes in *S. italica* [48]. In *A. thaliana*, the *cellulose synthase* superfamily includes ten *CesA* genes and thirty *Csl* genes [49]. Among these genes involved in the biosynthesis of cellulose, we detected *A. donax* transcripts homologous to nine out of thirteen *CesA* genes and two to *Csl* genes of *S. italica*, as well as three genes of the *A. thaliana Ces* superfamily (Table 5), confirming the high homology of *A. donax* to *S. italica*.

**Table 5:** Comparison between *A. donax* transcriptome and *S. italica* and *A. thaliana* transcripts for cellulose biosynthesis

Gene symbol	Gene name	Accession no.^a^	Species	No. of *A. donax* transcripts
*CesA1*	*Cellulose synthase A1*	Seita.5G122700.1	*S. italica*	7
*CesA2*	*Cellulose synthase A2*	AT4G39350.1	*A. thaliana*	6
*CesA2*	*Cellulose synthase A2*	Seita.4G211600.1	*S. italica*	4
*CesA3*	*Cellulose synthase A3*	Seita.2G115400.1	*S. italica*	6
*CesA4*	*Cellulose synthase A4*	Seita.9G227400.1	*S. italica*	1
*CesA5*	*Cellulose synthase A5*	Seita.9G020600.1	*S. italica*	6
*CesA6*	*Cellulose synthase A6*	AT5G64740.1	*A. thaliana*	7
*CesA6*	*Cellulose synthase A6*	Seita.3G332300.1	*S. italica*	4
*CesA8*	*Cellulose synthase A8*	Seita.5G319100.1	*S. italica*	3
*CslB1*	*Cellulose synthase*-*like B1*	Seita.1G268900.1	*S. italica*	3
*CslD1*	*Cellulose synthase*-*like D1*	AT2G33100.2	*A. thaliana*	2
*CslE6*	*Cellulose synthase*-*like E6*	Seita.2G243900.1	*S. italica*	2

A total of nine *S. italica* transcripts and three *A. thaliana* transcripts involved in cellulose biosynthesis showed homology with *A. donax* transcripts

^a^Phytozome (v11.0) accession numbers

For most genes, of both *S. italica* and *A. thaliana,* multiple homologous leaf transcripts of *A. donax* were retrieved, with a maximum of seven copies for *CesA1*, homologous to *S. italica*, and *CesA6*, homologous to *A. thaliana.* In line with the high homology of *A. donax* to the *S. italica* cellulose biosynthesis pathway, all ten lignin biosynthesis genes known for *S. italica* were also detected in the *A. donax* transcriptome (Table 6). Again, high copy numbers were detected for key genes in the lignin biosynthesis pathway, such as for *phenylalanine ammonia*-*lyase* (*PAL*) (13 copies) and *trans*-*cinnamate 4*-*hydroxylase* (*C4H*) (8 copies). These high copy numbers further stress the importance of key genes in biomass composition in *A. donax*.

**Table 6:** Comparison between *A. donax* transcriptome and *S. italica* transcripts for lignin biosynthesis

Gene symbol	Gene name	Accession no.^a^	Species	No. of *A. donax* transcripts
*PAL*	*Phenylalanine ammonia*-*lyase*	Seita.1G240400.1	*S. italica*	13
*CCoAOMT*	*Caffeoyl*-*CoA 3*-*O*-*methyl*-*transferase*	Seita.6G197700.1	*S. italica*	1
*CCR*	*Cinnamoyl*-*CoA reductase*	Seita.2G256200.1	*S. italica*	1
*CAD*	*Hydroxycinnamyl alcohol dehydrogenase*	Seita.1G057300.1	*S. italica*	1
*COMT*	*Caffeate O*-*methyltransferase*	Seita.6G059400.1	*S. italica*	3
*F5H*	*Ferulate 5*-*hydroxylase*	Seita.9G193900.1	*S. italica*	5
*HCT*	*Hydroxycinnamoyl transferase*	Seita.7G155700.1	*S. italica*	7
*C3H*	*Trans*-*cinnamate 3*-*hydroxylase*	Seita.3G194300.1	*S. italica*	6
*C4H*	*Trans*-*cinnamate 4*-*hydroxylase*	Seita.5G361200.1	*S. italica*	8
*4CL*	*4 coumarate CoA*-*ligase*	Seita.6G093400.1	*S. italica*	3

A total of ten *S. italica* transcripts involved in lignin biosynthesis showed homology with *A. donax* transcripts

^a^Phytozome (v11.0) accession numbers

We further assessed the presence of *A. donax* leaf transcripts homologous to *S. italica* and *A. thaliana* genes involved in stomatal development and distribution (Table 7), key genes in the response to drought stress. Five of the seven genes [*erecta* (*ER*), *epidermal patterning factor*-*like protein 9* (*EPLF9/STOMAGEN*), *erecta*-*like1* (*ERL1*), *GT2*-*like1* (*GTL1*), *FMA/bHLH097* (*FAMA*)] known to be involved in stomatal development in *S. italica* showed sequence similarity with *A. donax* leaf transcripts, whereas no sequence similarity was detected between *A. donax* transcripts and *A. thaliana* genes.

**Table 7:** Comparison between *A. donax* transcriptome and *S. italica* transcripts for stomatal development and distribution

Gene symbol	Gene name	Accession no.^a^	Species	No. of *A. donax* transcripts
*ER*	*Erecta*	Seita.4G086700.1	*S. italica*	5
*EPLF9/STOMAGEN*	*Epidermal patterning factor*-*like protein 9*	Seita.5G425700.1	*S. italica*	1
*GTL1*	*GT2*-*like1*	Seita.7G184400.1	*S. italica*	1
*ERL1*	*Erecta*-*like1*	Seita.4G019700.1	*S. italica*	2
*FAMA*	*FMA/bHLH097*	XM_004961002.3	*S. italica*	5

A total of six *S. italica* transcripts involved in stomatal development and distribution showed homology with *A. donax* transcripts

^a^ Phytozome (v11.0) and NCBI accession numbers

SAPs are fast emerging as potential gene family candidates for systems and synthetic biology approaches to improve multiple abiotic stress tolerance in plants [50]. Thus, they would be invaluable in the improvement of bioenergy crops such as *A. donax*. So far, however, until relatively recently, such genes had only been identified in few plant species (for example, in rice and sorghum). Therefore, we investigated the homology of *A. donax* leaf transcripts to genes encoding SAPs not only in the *A. thaliana* and *S. italica*, but also in *O. sativa* and *S. bicolor* transcript catalogs (Table 8).

**Table 8:** Comparison between *A. donax* transcriptome and *O. sativa* and *S. bicolor* transcripts for SAPs

Gene symbol	Gene name	Accession no.^a^	Species	No. of *A. donax* transcripts
*SAP1*	*Stress-associated protein 1*	LOC_Os09g31200.1	*O. sativa*	1
*SAP4*	*Stress-associated protein 4*	LOC_Os02g10200.1	*O. sativa*	1
*SAP4*	*Stress-associated protein 4*	Sobic.004G079100.2	*S. bicolor*	3
*SAP5*	*Stress-associated protein 5*	Sobic.002G245800.2	*S. bicolor*	2
*SAP8*	*Stress-associated protein 8*	LOC_Os06g41010.1	*O. sativa*	3
*SAP9*	*Stress-associated protein 9*	Sobic.002G046100.1	*S. bicolor*	1
*SAP9*	*Stress-associated protein 9*	LOC_Os07g07350.1	*O. sativa*	1
*SAP11*	*Stress-associated protein 11*	Sobic.002G345300.2	*S. bicolor*	3
*SAP11*	*Stress-associated protein 11*	LOC_Os08g39450.1	*O. sativa*	1
*SAP15*	*Stress-associated protein 15*	LOC_Os05g23470.1	*O. sativa*	1
*SAP16*	*Stress-associated protein 16*	LOC_Os07g38240.1	*O. sativa*	3

A total of seven *O. sativa* transcripts and four *S. bicolor* transcripts encoding for SAPs showed homology with *A. donax* transcripts

^a^Phytozome (v11.0) accession numbers

We identified *A. donax* transcripts homologous to seven of the 18 genes encoding for *O. sativa* SAPs, and to four of the *S. bicolor* SAPs. In addition, no sequence similarity was detected between *A. donax* transcripts and those of *S. italica* and *A. thaliana*.

### Genic-SSR marker mining, distribution, and polymorphism prediction

Many of the sequenced mRNA transcripts contained untranslated regions (UTRs) and occasional remaining introns. Such non-coding regions are prone to sequence diversity, which might be a source for potential polymorphic markers. Currently, no genetic markers are available for *A. donax*. Hence, we mined the retrieved transcripts for SSR markers, which are commonly used as a tool for assessing genetic diversity, the development of genetic maps, marker-assisted selection (MAS) breeding, and comparative genomics. The 62,596 unitranscripts, accounting for 52.7 Mbp, were screened for repeat motifs to explore the SSR profile in the *A. donax* leaf transcriptome. A total of 8364 SSRs were obtained from 7491 unitranscript sequences (11.97%) so 775 sequences contained more than one SSR. In addition, 294 sequences revealed compound SSR formation (Additional file 6: Table S3). A strong enrichment of mono- and tri-nucleotide repeat motifs was further detected among each of the SSR classes, while longer repeat motifs were clearly underrepresented (Additional file 6: Table S4). The complete list of unitranscripts with the description of the identified SSR motifs is reported in Additional file 7: Table S5, whereas the frequencies of each of the different SSR repeats as compared to the total identified SSRs are reported in Additional file 7: Table S6. We further investigated the distribution of SSR classes in the unitranscripts (CDS, 5′ UTR, and 3′ UTR). Out of the total 8364 SSRs, 5592 were located in the UTR and CDS regions, of which 611 (10.93%), 2059 (36.82%), and 2922 (52.25%) SSRs were located in the 5′ UTR, CDS, and 3′ UTR, respectively. The remaining SSRs (2772 SSRs; 33.14%) were not ascertained a position because it was not possible to delimit the UTR and CDS regions for the transcripts containing them. For the SSRs located in the CDS, 75.52% (1555 SSRs) were tri-nucleotides, followed by di-nucleotides (200 SSRs; 9.71%). Conversely, the proportion of tri-nucleotides within the UTR regions was lower compared to the CDS. Mono- (2609 SSRs; 82.48%) and tri- (303 SSRs; 9.58%) nucleotides were the most abundant in the 3′-UTR region, while in the 5′-UTR 457 (40.23%) and 420 (36.97%) SSRs were mono- and tri-nucleotides, respectively. With respect to tri-nucleotides within the CDS region, the CGC repeats (114 SSRs; 7.33%) were most represented within the 1555 tri-nucleotide motifs, followed by GGC (109 SSRs; 7.01%) and CGG (107 SSRs; 6.88%) repeats. Within the mono- and tri- nucleotides in the 5′-UTR region, T (246 SSRs; 53.83%), A (139 SSRs; 30.42%), CCG (30 SSRs; 7.14%), and GGC (30 SSRs; 7.14%) repeats were the most common. Similarly, among the mono- and tri-nucleotide SSR classes in the 3′-UTR, the highest frequencies were observed for T (1650 SSRs; 63.24%), A (848 SSRs; 32.50%), GGC (17 SSRs; 5.61%), and AGC (16 SSRs; 5.28%) repeats.

Finally, in order to detect PolySSRs, ecotype-specific assemblies were reconstructed using the Trinity software. The assembly statistics of the transcript dataset of each ecotype is reported in Additional file 8: Table S7. Using CandiSSR pipeline, a total of 53 PolySSRs were identified between *A. donax* unitranscripts (hereafter referred to as reference transcriptome) and the three ecotype-specific transcriptomes (Additional file 9: Table S8), suggesting the existence of a certain degree of genetic diversity among the three ecotypes analyzed in this study. The specific primer pairs for the 53 PolySSRs were designed using Primer3, which is implemented in the CandiSSR Perl script (Additional file 9: Table S9). The polymorphism encompassed di-nucleotide motifs with a minimum of six repeats and tri-nucleotide motifs with a minimum of five repeats in the reference transcriptome. The total number of PolySSR motifs ranged from 1 to 4 and the minimum and maximum number of repeats was 2 for AGC, GTT, and TCC motifs, and 11 for AG, respectively (Additional file 9: Table S10).

### Gene functions of the unitranscript sequences containing PolySSRs

To date, no gene-related markers have been identified in *A. donax*. We therefore conducted functional categorization of unitranscripts containing SSRs in order to reveal homology with known proteins and determine the possible functions of the 53 polymorphic genic-SSRs by BLAST2GO analysis with the NR database of protein sequences. A total of 31 GO terms were allocated to the transcripts containing the PolySSRs. They were assigned to the BP (157 sequences, 20 GOs), CC (132 sequences, 8 GOs), and MF (49 sequences, 3 GOs) categories. In addition, nineteen KEGG metabolic pathways were represented by at least one transcript with the corresponding EC numbers. Particularly, the phenylpropanoid biosynthesis, starch and sucrose metabolism, and purine and thiamine metabolisms were overrepresented.

Interestingly, functional annotation revealed that unitranscripts containing PolySSRs belonged to key genes involved in the hydrolysis of ABA glucose ester (ABA-GE) to free ABA (i.e., *β*-*glucosidase 10*) [51], in the positive regulation of ABA signaling pathway (i.e., *3*-*ketoacyl*-*CoA thiolase*) [52], and to several transcription factors (TFs) involved in enhancing drought tolerance in plants, such as myeloblastosis (MYB) TFs (i.e., MYB1R1) [53] and heat stress TFs (i.e., HSFA3) [54]. Furthermore, among the positive hits were genes known to be involved in photoperiod perception, circadian regulation, and regulation of flowering, such as *early flowering 4*-*like* genes (i.e., ELF4- like 4) [55], and in the *Agrobacterium tumefaciens*-mediated genetic transformation, such as *VirE2 interacting protein2* (i.e., *VIP2*) [56]. Furthermore, homologous sequences to *glutathione S*-*transferase* (*GST*) genes, whose detoxification and antioxidant activities can be considered important factors in drought tolerance in plants, such as in *H. vulgare* [57] and in transgenic *A. thaliana* [58] were also found among the unitranscripts containing PolySSRs. These findings make these functional PolySSRs particularly useful for research into accelerated genetic studies and enhanced breeding programmes in *A. donax*.

### Experimental validation of PolySSR markers

To test whether the potential SSR loci that were mined were true-to-type ones for use in genetic diversity studies, a total of five primer pairs were randomly selected from those obtained from the CandiSSR pipeline (i.e., forward and reverse primer pairs n.1 of CPSSR_1|AAG, CPSSR_3|AGA, CPSSR_4|AGC, CPSSR_5|AGC, and CPSSR_9|CAG; see Additional file 9: Table S9) and synthesized. The results showed that all tested primer pairs amplified successfully. Out of the five pairs of primer, four produced amplicons with expected sizes, while one primer pair gave an amplicon larger than the expected size (i.e., CPSSR_1|AAG). Consequently, the former pairs of primer were then used to assess the genetic diversity among the three *A. donax* ecotypes. Out of the four primer pairs, one (i.e., CPSSR_4|AGC) successfully amplified polymorphic SSR fragment; particularly, (AGC/GCT)_5_ repeats were detected in EcoA, whereas (AGC/GCT)_4_ repeats were identified in EcoB and EcoC (Additional file 10: Figure S3).

## Discussion

Although *A. donax* is one of the most promising and emerging non-food bioenergy crops in regions with Mediterranean climates, until very recently available genomics resources were scarce. The knowledge about the molecular mechanisms of *A. donax*, underlying its adaptability in marginal lands with low input requirements, its high biomass yield and tolerance to abiotic stresses, is still in its infancy due to the little information in public databases about *Arundo* transcriptome profiles. Therefore, the transcriptomic data generated in this study provide invaluable resources to understand the biology of *A. donax*, offering new tools and methodologies to help develop agriculturally productive and economically sound genotypes.

It is worth mentioning that genomes at species level are dynamic and are composed by genes which are present in each individual of a species (core genes) and genes in a set of individuals (dispensable genes); collectively, these genes constitute the so-called pan-genome. For example, transposable elements are largely responsible for the variation in both intergenic and genic regions, not only in closely related species but also between individuals within a species. These suggest that a single genome sequence might not reflect the entire genomic information of a species [59]. The presence of pan-genome and pan-transcriptome was observed also in many plant species. For instance, a study of the pan-genome was conducted by resequencing of six elite *Z. mais* inbred lines for commercial relevance in China and several hundreds of complete dispensable genes with presence/absence variation among these lines were identified [60]. More recently, a transcriptome sequencing of 503 maize inbred lines revealed the dynamic nature of the maize pan-genome and identified representative transcript assemblies (RTAs) with 16.4% expressed in all lines and 82.7% expressed in subsets of the lines, demonstrating that a considerable portion of variation may be outside the single reference genome for a species [61]. Also DNA and RNA sequencing of seven accessions of *Glycine soya* (wild soybean) revealed that approximately 80% of the pan-genome was present in all the accessions, representing the core genome, and the remaining were dispensable genes that exhibited greater variation reflecting a putative role in adaptation to different environments [62]. This reflects the importance of sequencing and assembling numerous *A. donax* transcriptomes in order to capture all the possible variable dispensable genes/transcripts and decipher their role in adaptation to different environments. Further analysis of the pan-transcriptome rather than a single reference circumvents single sample bias and ensures that the majority of genetic diversity within *A. donax* is fully captured. Furthermore, de novo construction of *A. donax* pan-transcriptome is also necessary to unravel variation in gene regulation and expression and provide additional candidate genes for the development of elite genotypes.

De novo RNA-Seq assembly facilitates the study of transcriptomes for plant species without sequenced genomes, but it is challenging to select the most accurate assembly in this context. Here, we developed a specialized bioinformatics workflow for de novo transcriptome assembly. Illumina next-generation mRNA-Seq was successfully used to assemble and analyze the leaf transcriptome profile of three *A. donax* ecotypes representing a vast array of geographical adaptation across the species and possessing traits of agronomic importance grown under two different field conditions in order to increase the understanding of biological processes and shed new light on the molecular mechanisms underlying the outstanding adaptability of this relatively unknown species.

More than 1252 million of single-end transcript reads were generated by Illumina sequencing. Using a two-step procedure along with single and multiple *k*-*mer* methods, we de novo assembled 62,596 unitranscripts based on 1249 million of filtered reads. The number of retrieved leaf transcripts was lower than the number of transcripts obtained in previous transcriptome reconstructions in *A. donax* [10–12], but it was similar to those recently obtained in *Lolium* species (*L. multiforum*: 55,570 sequences; *L. multiforum var. westerwoldicum*: 52,166 sequences; *L. temulentum*: 59,309 sequences) [63]. This was most probably due to the clustering of the different pre-assemblies by CD-HIT and the redundancy removal using the tr2aacds pipeline to obtain the final assembly.

The N50 length of the unitranscripts was 1134 bp, and the average length was 842 bp (Table 1); these results are comparable to recently published plant transcriptomes, such as common reed (*Phragmites australis*; N50 = 1187 bp, average length = 642 bp) [64], common wild rice (*O. rufipogon* Griff.; N50 = 1147 bp, average length = 715 bp) [65], and sugarcane (*Saccharum officinarum*; N50 = 1177 bp) [66]. Additionally, the GC content of the assembled unitranscripts (49%) was slightly higher compared to those obtained previously in *A. donax* (45.60% [10] and 47.70%; [11]), but it was similar to that observed in *P. australis* using Illumina sequencing (49.9%) [64].

To evaluate assembly consistency and completeness, the percentage of reads mapping back to the assembled leaf transcriptome was assessed (Table 2). The observed percentage of mapped reads was about 71% and this result is similar with that retrieved in *T. aestivum* [67] and in *T. turgidum* [68].

We further assessed how much overlap there was between *A. donax* unitranscripts and those from related species (*H. vulgare*, *T. aestivum*, *S. bicolor*, *O. sativa*, and *S. italica*). Similarly to *Lolium* species [63], the highest number of hits was obtained for *H. vulgare* (6526 transcripts with 100% coverage and 11,443 transcripts with >70% coverage) and *T. aestivum* (6503 transcripts with 100% coverage and 12,385 transcripts with >70% coverage) (Table 3). We also checked the completeness of our transcriptome assembly using BUSCO quality assessment tool. The quality of the *A. donax* leaf transcriptome was comparable to or better than those of the majority of transcriptome assemblies listed in [30] and the *Spinacia oleracea* transcriptome assembly [69].

Although our study is based only on one vegetative tissue (leaf), it has broadened our knowledge of the transcriptome profile in *A. donax* grown under natural conditions. About 81% and 55% of the unitranscripts and predicted proteins were successfully assigned to genes by BLASTx and BLASTp searches against the UniRef90 database (Table 4). Moreover, a lower number of BLASTx (54%) and BLASTp (34%) matches were obtained with the UniProtKB/Swiss-Prot database, but this result was similar to that observed in *S. officinarum* [66]. Pfam domains and signal peptides were assigned to 32% and 2.76% of the identified proteins, respectively; these numbers are lower compared to those obtained in previous studies in perennial ryegrass (*Lolium perenne*) [70], *Lolium* and *Festuca* species (i.e., *F. pratensis*; *L. multiflorum*; *L. m. westerwoldicum*; *L. temulentum*) [63], and sugarcane (*S. officinarum*) [66].

The Blast2GO suite using the NCBI NR protein database was also adopted to annotate the *A. donax* leaf transcriptome. A lower number of BLAST hits (26,323) against this database were obtained compared to the UniRef90 and UniProtKB/Swiss-Prot databases. As expected, a considerable sequence similarity was observed with the related species *S. italica*, *S. bicolor*, *Z. mays*, and *O. sativa japonica* (Fig. 3). We retrieved 820 GO terms, and of the three main GO categories, 63% of GO terms were assigned to BPs, about 18% were assigned to MFs, and 13% to CCs. Moreover, three subcategories (metabolic processes, biosynthetic processes, and cellular processes) were enriched in the BP category, two subcategories (binding proteins and proteins with catalytic activities) in the MF category and four subcategories (cell, cell part, organelle, and membrane) in the CC category (Fig. 4). A high proportion of unitranscripts showed no similarity to any known putative protein in public databases, suggesting that these transcripts could possibly be present due to a combination of factors, such as the presence of long non-coding RNAs, untranslated regions, genes without any annotation information or lack of known protein domains, and finally novel genes in *A. donax* that require more experimentation to be characterized in detail. For example, transcripts without BLAST matches were observed previously in plant transcriptome reconstruction in celery (*Apium graveolens*) [71], in perennial ryegrass (*L. perenne*) [70], and in sugarcane (*S. officinarum*) [72]. However, future studies will aim to identify and functionally characterize long non-coding RNAs in *A. donax* transcriptome.

Biological pathway studies play a pivotal role in gaining insight into functional analysis and interpretation of transcriptomic data. KEGG is a highly integrated database that allows the comprehensive interrogation of an organism’s genome content [73]. Here, we analyzed purine (Fig. 5a) and thiamine (Fig. 5b) metabolism, phenylpropanoid biosynthesis (Fig. 6a; Table 6), starch and sucrose metabolism (Fig. 6b), cellulose biosynthesis (Table 5), carbon fixation (Additional file 5: Figure S2), stomatal development and distribution pathways (Table 7), and finally the presence of homologous transcripts encoding for SAPs (Table 8).

Purine metabolism, particularly adenine metabolism, has been reported for *Arabidopsis oxt1* mutants to be involved in activating plant abiotic defenses. The cellular adenine level plays a crucial role in signals that control abiotic stress responses and plant growth, indicating a connection between purine metabolism, plant growth responses, and stress acclimation [47]. Moreover, allantoin, a metabolic intermediate of purine catabolism, often accumulates in plants subjected to abiotic stress. Particularly, the role of allantoin in the activation of jasmonic acid responses via ABA has been recently reported for *Arabidopsis* knockout mutants (*aln*), suggesting a possible link of purine catabolism with stress phytohormone homeostasis and signaling [74]. Furthermore, thiamine (vitamin B_1_) and its phosphate esters act as cofactors in response to abiotic and biotic stress in plants [75]. Interestingly, its metabolism can be altered in plants under environmental stress, e.g., in *Z. mays* under abiotic stress conditions [76].

Additionally, several putative targets for lignocellulosic biomass production and improvement were evaluated in this study. A prevalent proportion of plants biomass is composed of cell wall polymers; in secondary walls, cellulose is the load-bearing unit that cross-links with hemicelluloses (xylan and glucomannan) and, together with lignin, this complex network forms the secondary cell wall, an important source for biofuel production. Lignins are complex racemic aromatic heteropolymers that are products of the phenylpropanoid metabolism, whereas cellulose is made of *β*-1,4-linked glucosyl residues and its biosynthesis is mediated by cellulose synthase complexes, called rosettes, located in the plasma membrane. The proportion of these components differs between different plant species and environmental stimuli [77]. In the *A. donax* leaf transcriptome, many homologous transcripts encoding for lignins and cellulose were retrieved.

The analysis of the carbon fixation pathway revealed, as expected, that the majority of the metabolic genes of this pathway are expressed in *A. donax* leaves. It has long been known that the C fixed during photosynthesis is transformed into sugar or sugar derivatives in photosynthetic source cells. These compounds are then transported by the phloem to sink tissues where it is consumed by cells to obtain energy and building blocks for growth or stored in vacuoles, as polymers (starch or plastids) and as structural molecules (cellulose, hemicellulose, and lignin) [78]. In sugarcane, sucrose synthesized in leaves is deposited both inside and outside of stalk (culm) parenchyma cells and in secondary cell wall components, particularly as cellulose [79]. Consequently, improving the production of photosynthates, which are subsequently accumulated in polymers and structural molecules, could be a strategy for the success of *A. donax* as a bioenergy crop.

Stomatal development, distribution, and aperture mechanisms are finely regulated in plants in order to control transpiration and water use efficiency (WUE) under different environmental conditions. In the *A. donax* leaf transcriptome, five homologous transcripts were retrieved. Particularly, the detection of the *GTL1* protein as a transcriptional repressor of *SDD1*, negatively regulating stomatal density in order to regulate WUE [80], is an important finding. The presence of *GTL1* in the assembled unitranscripts might be associated to an adaptive response of *A. donax* that prevents water loss in dry conditions.

The presence of homologs encoding for SAPs was investigated in the *A. donax* leaf unitranscripts. These A20/AN1 zinc-finger proteins were reported as an important component of stress responses in rice, where 18 SAP encoding genes were inducible by multiple abiotic stresses such as cold, salt, and dehydration stresses [81].

The presence of homologous transcripts to genes involved in the abovementioned pathways provides new insights into molecular mechanisms underlying the extreme adaptability and robustness of *A. donax* to a wide range of environmental stresses, thereby contributing to its importance as a bioenergy crop.

Finally, for the first time SSR markers were identified in the *A. donax* leaf transcriptome. Using the MISA software, a total of 8364 genic-SSRs were obtained from 7491 sequences. In this SSRs catalogue, the most frequent repeat type was the mono-nucleotide motif with 4170 SSRs (49.86%), followed by tri-nucleotide repeats with 3034 SSRs (36.27%) and di-nucleotide repeats with 1060 SSRs (12.67%). This distribution frequency differs from those previously reported in sugarcane [72] in which the most frequent SSR motif is the tri-nucleotide repeat. Genome-wide SSR analyses in nine species of the Poaceae family [82] revealed that the most abundant repeat type was a hexamer (58.82%). Conversely, our results were consistent with those obtained in licorice (*Glycyrrhiza uralensis*) in which the most frequent repeat motif was a mono-nucleotide, followed by tri-nucleotides and di-nucleotide motifs [83], and partially to those obtained in bhumyamalaki (*Phyllanthus amarus*), in which the most abundant repeat type was a mono-nucleotide, followed by di- and tri-nucleotides [84]. Although natural genetic variation in *A. donax* was reported to be low for the Mediterranean basin and slightly higher for the Asian area [85], and genetic uniformity was also observed in ecotypes in the United States [86], here 53 Candidate PolySSRs were predicted using CandiSSR software, suggesting a likely high level of genetic diversity among the analyzed ecotypes. To test whether the identified PolySSRs were true-to-type ones for use in genetic diversity studies, we evaluated a total of five primer pairs, and one pair resulted to amplify a polymorphic fragment among the three ecotypes (Additional file 10: Figure S3), suggesting that the PolySSRs that were mined using the CandiSSR pipeline represent a valuable resource for assessing the genetic variability in *A. donax*. As shown in Additional file 9: Table S8, for the CPSSR_4 PolySSR no transcripts containing the polymorphic fragment were detected in EcoA and EcoB. This is probably due to the lack or partial reconstruction of sequences containing the repeat motif in the transcriptomes of EcoA and EcoB. The use of identified SSR markers will help in the exploitation of variation at genomic and expression levels, in the analysis of genetic diversity in future *A. donax* genomic studies, and in the identification of ecotypes for commercial utilization. Particularly, the set of PolySSRs identified in this study will facilitate the assessment of the polymorphic status in other *A. donax* ecotypes, by easing the costly and time-consuming traditional laboratory assessment of SSR development.

In addition, we found that the majority of genic-SSRs are present in functional genes, indicating that these markers could possibly be associated with phenotypes of agricultural value. SSRs located in coding and untranslated regions (transcribed SSRs) can be efficient functional markers in genic regions [17]. The results of this study showed that SSR markers in *A. donax* leaf transcriptome did not distribute evenly in CDS and UTR regions, with more occurrence in the latter than in the former. This was consistent with the results of other transcriptome studies [87–90]. By further studying these differences in microsatellites density and their distribution pattern in the *A. donax* transcriptome, more precise inferences about their function can be made. As mutations lead to the alteration of microsatellite length through the addition or deletion of repeat units, and since SSRs are more prone to indel mutations by means of slipped-strand mispairing [91], their prevalence in the CDS region is more likely to alter gene function (gain or loss) as compared to the UTR regions. Nevertheless, microsatellites in UTRs exhibit higher tolerance for mutations and as a result they may possess the tuning ability on functional genes [92, 93], conferring the genes adaptive roles in evolution [94]. The functional categorization of *A. donax* unitranscripts containing SSRs revealed that these transcripts represent several key transcribed genes with biology, cellular, and molecular functions. Therefore, while allowing studies on genetic variation, SSR markers derived from transcripts also provide information on gene function related to possible phenotypic differences between the *A. donax* ecotypes. Newly designed SSR primers for *A. donax* can be used to detect SSRs in other species in Poaceae, and explore their transferability to other closely related species that also lack genomic resources.

## Conclusions

In the current study, we employed de novo RNA-Seq assembly to investigate and analyze the *A. donax* leaf transcriptomic profile. Currently, the data reported here provide the first publicly available comprehensive leaf transcriptome resource for *A. donax*. To facilitate molecular research in *A. donax*, we functionally annotated and characterized 62,596 leaf unitranscripts. We further assessed the occurrence of genes involved in crucial metabolic pathways, thereby providing new insight into the molecular mechanisms underlying the extreme adaptability of *A. donax* to different environmental conditions and its importance as a bioenergy crop. Further, this study provides the first SSR marker catalogue and a set of PolySSRs for *A. donax*, which will be a useful resource for future genetic and genomic studies in this still poorly investigated species. The genomic data herein generated for *A. donax* ecotypes represent a counterpart to the genomic resources already available for this species. Altogether, this dataset will facilitate advancements in genetic and molecular studies and allow the implementation of breeding strategies for this important bioenergy crop.

## Additional files

**Additional file 1.** Description of water treatments imposition, soil water status, and meteorological conditions.

**Additional file 2: Figure S1.** Soil water status and meteorological conditions at the experimental site in 2014 observed from DOY 152 to DOY 272. (a) Daily maximum (red line), mean (black line), and minimum (blue line) air temperature (T_air_, °C) are shown. (b) Daily rainfall (Rainfall, mm) and water deficit (Wd, mm) are shown as black and gray bars, respectively. The positive values of Wd are not reported. (c) Soil volumetric water content (θ), expressed as percentage of the field capacity (θ_fc_), is shown as black solid and black dashed lines in WW and in mDr, respectively.

**Additional file 3: Table S1.** Distribution of gene ontology (GO) terms among biological process, molecular functions and cellular components categories using Blast2GO.

**Additional file 4: Table S2.** Kyoto Encyclopedia of Genes and Genomes (KEGG) pathways analysis for *A. donax* leaf transcriptome.

**Additional file 5: Figure S2.** Carbon fixation pathway genes found in *A. donax* leaf transcriptome are depicted by the different colored ECs (one color for each EC).

**Additional file 6: Table S3.** Frequency and distribution of the identified simple sequence repeat (SSR) classes in *A. donax* leaf transcriptome. **Table S4.** Frequency of SSR classes in the *A. donax* leaf transcriptome.

**Additional file 7: Table S5.** Description of the identified simple sequence repeat (SSR) types for the *A. donax* leaf transcriptome. **Table S6.** SSR repeat motifs in *A. donax* unitranscripts.

**Additional file 8: Table S7.** Summary statistics of each ecotype-specific assemblies.

**Additional file 9: Table S8.** Candidate PolySSRs identified in the reference and in the ecotype-specific assemblies. **Table S9.** Specific primer pairs designed for the 53 PolySSRs using Primer3 in the CandiSSR pipeline. **Table S10.** PolySSR motifs in the reference and ecotype-specific transcriptomes.

**Additional file 10: Figure S3.** Electropherogram analysis of the CPSSR_4 PolySSR in the three *A. donax* ecotypes. Electropherograms of the sequenced fragments of EcoA (**a**), EcoB (**b**) and EcoC (**c**).

## Abbreviations

BLAST: Basic Local Alignment Search Tool
BP: biological process
BUSCO: Benchmarking Universal Single-Copy Orthologs
BWA: Burrows–Wheeler alignment
CC: cellular component
EC: enzyme code
GO: gene ontology
GR_protein: Gramene Proteins Database
IPS: InterProScan
KEGG: Kyoto Encyclopedia of Genes and Genomes
MF: molecular function
MK: multi-*k*-*mer*
NCBI: National Center for Biotechnology Information
NGS: next-generation sequencing
NR: non-redundant
ORFs: open reading frames
PDB: protein data bank
PolySSRs: polymorphic SSRs
RMBT: reads mapping back to transcriptome
RNA-Seq: mRNA sequencing
RTAs: representative transcript assemblies
SAPs: stress-associated proteins
SE: single-end
SK: single-*k*-*mer*
SRA: short read archive
SSR: simple sequence repeat
TAIR: *Arabidopsis* Information Resource
TSA: TRANSCRIPTOME SHOTGUN ASSEMBLY
UniParc: UniProt Archive
UniProtKB/Swiss-Prot: Swiss-Prot section of UniProt KnowledgeBase
UniRef90: UniProt reference clusters

## References

[1] Hardion L, Verlaque R, Saltonstall K, Leriche A, Vila B (2014). Origin of the invasive *Arundo donax* (Poaceae): a trans-Asian expedition in herbaria. Ann Bot.

[2] Corno L, Pilu R, Adani F (2014). *Arundo donax* L. A non-food crop for bioenergy and bio-compound production. Biotechnol Adv.

[3] Balogh E, Herr JM, Czakó M, Márton L (2012). Defective development of male and female gametophytes in *Arundo donax* L. (Poaceae). Biomass Bioenergy.

[4] Xia Z, Xu H, Zhai J, Li D, Luo H, He C, Huang X (2011). RNA-Seq analysis and de novo transcriptome assembly of *Hevea brasiliensis*. Plant Mol Biol.

[5] Gross SM, Martin JA, Simpson J, Abraham-Juarez MJ, Wang Z, Visel A (2013). De novo transcriptome assembly of drought tolerant CAM plants, *Agave deserti* and *Agave tequilana*. BMC Genom.

[6] Wu T, Luo S, Wang R, Zhong Y, Xu X, Lin YE (2014). The first Illumina-based de novo transcriptome sequencing and analysis of pumpkin (*Cucurbita moschata* Duch.) and SSR marker development. Mol Breed.

[7] Du F, Wu Y, Zhang L, Li XW, Zhao XY, Wang WH (2015). De novo assembled transcriptome analysis and SSR marker development of a mixture of six tissues from *Lilium* Oriental hybrid ‘Sorbonne’. Plant Mol Biol Rep.

[8] Jia X, Deng Y, Sun X, Liang L, Ye X (2015). Characterization of the global transcriptome using Illumina sequencing and novel microsatellite marker information in seashore paspalum. Genes Genom.

[9] Martin LB, Fei Z, Giovannoni JJ, Rose JK (2013). Catalyzing plant science research with RNA-seq. Front Plant Sci.

[10] Sablok G, Fu Y, Bobbio V, Laura M, Rotino GL, Bagnaresi P (2014). Fuelling genetic and metabolic exploration of C_3_ bioenergy crops through the first reference transcriptome of *Arundo donax* L.. Plant Biotechnol J.

[11] Fu Y, Poli M, Sablok G, Wang B, Liang Y, Porta N (2016). Dissection of early transcriptional responses to water stress in *Arundo donax* L. by unigene-based RNA-seq. Biotechnol Biofuels.

[12] Barrero RA, Guerrero FD, Moolhuijzen P, Goolsby JA, Tidwell J, Bellgard SE (2015). Shoot transcriptome of the giant reed, *Arundo donax*. Data Br.

[13] Garg R, Shankar R, Thakkar B, Kudapa H, Krishnamurthy L, Mantri N (2016). Transcriptome analyses reveal genotype-and developmental stage-specific molecular responses to drought and salinity stresses in chickpea. Sci Rep.

[14] Carrington JC, Ambros V (2003). Role of microRNAs in plant and animal development. Science.

[15] Miguel A, de Vega-Bartol J, Marum L, Chaves I, Santo T, Leitão J (2015). Characterization of the cork oak transcriptome dynamics during acorn development. BMC Plant Biol.

[16] Zhao Y, Williams R, Prakash CS, He G (2013). Identification and characterization of gene-based SSR markers in date palm (*Phoenix dactylifera* L.). BMC Plant Biol.

[17] Li YC, Korol AB, Fahima T, Nevo E (2004). Microsatellites within genes: structure, function, and evolution. Mol Biol Evol.

[18] FastQC: a quality control tool for high throughput sequence data. 2015. http://www.bioinformatics.babraham.ac.uk/projects/fastqc/. Accessed 12 Aug 2015.

[19] FASTX-Toolkit: fastq/a short-reads pre-processing tools. 2014. http://hannonlab.cshl.edu/fastx_toolkit/index.html. Accessed 2 Oct 2015.

[20] Grabherr M, Haas B, Yassour M, Levin JZ, Thompson DA, Amit I (2011). Trinity: reconstructing a full-length transcriptome without a genome from RNA-Seq data. Nat Biotechnol.

[21] Robertson G, Schein J, Chiu R, Corbett R, Field M, Jackman SD (2010). De novo assembly and analysis of RNA-seq data. Nat Methods.

[22] rnaSPAdes: de novo RNA-Seq assembler. http://bioinf.spbau.ru/en/rnaspades. Accessed 12 Nov 2015.

[23] Fu L, Niu B, Zhu Z, Wu S, Li W (2012). CD-HIT: accelerated for clustering the next-generation sequencing data. Bioinformatics.

[24] EvidentialGene: tr2aacds, mRNA transcript assembly software. 2013. http://arthropods.eugenes.org/EvidentialGene/about/EvidentialGene_trassembly_pipe.html. Accessed 15 Dec 2015.

[25] Langmead B, Salzberg SL (2012). Fast gapped-read alignment with Bowtie 2. Nat Methods.

[26] Li H, Durbin R (2009). Fast and accurate short read alignment with Burrows-Wheeler transform. Bioinformatics.

[27] Li H, Handsaker B, Wysoker A, Fennell T, Ruan J, Homer N (2009). The sequence alignment/map format and SAMtools. Bioinformatics.

[28] EnsemblPlants FTP server. 2015. http://ftp.ensemblgenomes.org/pub/plants/release-29/fasta/. Accessed 5 Nov 2015.

[29] RNA-Seq de novo assembly using Trinity. 2015. https://github.com/trinityrnaseq/trinityrnaseq/wiki. Accessed 13 Aug 2015.

[30] Simão FA, Waterhouse RM, Ioannidis P, Kriventseva EV, Zdobnov EM (2015). BUSCO: assessing genome assembly and annotation completeness with single-copy orthologs. Bioinformatics.

[31] TransDecoder (find coding regions within transcripts). 2014. http://transdecoder.github.io/. Accessed 14 Jan 2016.

[32] Trinotate: transcriptome functional annotation and analysis. 2014. http://trinotate.github.io. Accessed 14 Jan 2016.

[33] The Broad Institute. 2015. https://data.broadinstitute.org/Trinity/Trinotate_v2.0_RESOURCES/. Accessed 14 Jan 2016.

[34] Finn RD, Clements J, Eddy SR (2011). HMMER web server: interactive sequence similarity searching. Nucleic Acids Res.

[35] Petersen TN, Brunak S, von Heijne G, Nielsen H (2011). Signalp 4.0: discriminating signal peptides from transmembrane regions. Nat Methods.

[36] Conesa A, Gotz S, Garcia-Gomez JM, Terol J, Talon M, Robles M (2005). Blast2GO: a universal tool for annotation, visualization and analysis in functional genomics research. Bioinformatics.

[37] Matsunaga A, Tsugawa M, Fortes J. Cloudblast: Combining mapreduce and virtualization on distributed resources for bioinformatics applications. IEEE Fourth International Conference on eScience; 2008.

[38] MISA—MIcroSAtellite identification tool. 2002. http://pgrc.ipk-gatersleben.de/misa/. Accessed 8 Mar 2016.

[39] Xia EH, Yao QY, Zhang HB, Jiang JJ, Zhang LP, Gao LZ (2016). CandiSSR: an efficient pipeline used for identifying candidate polymorphic SSRs based on multiple assembled sequences. Front Plant Sci.

[40] Koressaar T, Remm M (2007). Enhancements and modifications of primer design program Primer3. Bioinformatics.

[41] Untergasser A, Cutcutache I, Koressaar T, Ye J, Faircloth BC, Remm M (2012). Primer3—new capabilities and interfaces. Nucleic Acids Res.

[42] BioEdit—biological sequence alignment Editor. (http://www.mbio.ncsu.edu/bioedit/bioedit.html). Accessed 5 Dec 2016.

[43] Nakasugi K, Crowhurst R, Bally J, Waterhouse P (2014). Combining transcriptome assemblies from multiple de novo assemblers in the allo-tetraploid plant *Nicotiana benthamiana*. PLoS ONE.

[44] Miller JR, Koren S, Sutton G (2010). Assembly algorithms for next-generation sequencing data. Genomics.

[45] Cantu D, Pearce SP, Distelfeld A, Christiansen MW, Uauy C, Akhunov E (2011). Effect of the down-regulation of the high *Grain Protein Content* (*GPC*) genes on the wheat transcriptome during monocarpic senescence. BMC Genom.

[46] He B, Zhao S, Chen Y, Cao Q, Wei C, Cheng X, Zhang Y (2015). Optimal assembly strategies of transcriptome related to ploidies of eukaryotic organisms. BMC Genom.

[47] Sukrong S, Yun KY, Stadler P, Kumar C, Facciuolo T, Moffatt BA (2012). Improved growth and stress tolerance in the *Arabidopsis oxt1* mutant triggered by altered adenine metabolism. Mol Plant.

[48] Muthamilarasan M, Khan Y, Jaishankar J, Shweta S, Lata C, Prasad M (2015). Integrative analysis and expression profiling of secondary cell wall genes in C_4_ biofuel model *Setaria italica* reveals targets for lignocellulose bioengineering. Front Plant Sci.

[49] Richmond TA, Somerville CR (2000). The cellulose synthase superfamily. Plant Physiol.

[50] Giri J, Dansana PK, Kothari KS, Sharma G, Vij S, Tyagi AK (2013). SAPs as novel regulators of abiotic stress response in plants. BioEssays.

[51] Wang P, Liu H, Hua H, Wang L, Song CP (2011). A vacuole localized β-glucosidase contributes to drought tolerance in *Arabidopsis*. Chin Sci Bull.

[52] Jiang T, Zhang XF, Wang XF, Zhang DP (2011). *Arabidopsis* 3-ketoacyl-CoA thiolase-2 (KAT2), an enzyme of fatty acid β-oxidation, is involved in ABA signal transduction. Plant Cell Physiol.

[53] Baldoni E, Genga A, Cominelli E (2015). Plant MYB transcription factors: their role in drought response mechanisms. Int J Mol Sci.

[54] Guo M, Liu JH, Ma X, Luo DX, Gong ZH, Lu MH (2016). The plant heat stress transcription factors (HSFs): structure, regulation, and function in response to abiotic stresses. Front Plant Sci.

[55] Kolmos E, Nowak M, Werner M, Fischer K, Schwarz G, Mathews S (2009). Integrating *ELF4* into the circadian system through combined structural and functional studies. HFSP J.

[56] Anand A, Krichevsky A, Schornack S, Lahaye T, Tzfira T, Tang Y (2007). *Arabidopsis* VIRE2 INTERACTING PROTEIN2 is required for *Agrobacterium* T-DNA integration in plants. Plant Cell.

[57] Rezaei MK, Shobbar ZS, Shahbazi M, Abedini R, Zare S (2013). Glutathione S-transferase (GST) family in barley: identification of members, enzyme activity, and gene expression pattern. J Plant Physiol.

[58] Xu J, Xing XJ, Tian YS, Peng RH, Xue Y, Zhao W, Yao QH (2015). Transgenic *Arabidopsis* plants expressing tomato glutathione S-transferase showed enhanced resistance to salt and drought stress. PLoS ONE.

[59] Morgante M, De Paoli E, Radovic S (2007). Transposable elements and the plant pan-genomes. Curr Opin Plant Biol.

[60] Lai J, Li R, Xu X, Jin W, Xu M, Zhao H (2010). Genome-wide patterns of genetic variation among elite maize inbred lines. Nat Genet.

[61] Hirsch CN, Foerster JM, Johnson JM, Sekhon RS, Muttoni G, Vaillancourt B (2014). Insights into the maize pan-genome and pan-transcriptome. Plant Cell.

[62] Li YH, Zhou G, Ma J, Jiang W, Jin LG, Zhang Z (2014). De novo assembly of soybean wild relatives for pan-genome analysis of diversity and agronomic traits. Nature biotechnol.

[63] Czaban A, Sharma S, Byrne SL, Spannagl M, Mayer KF, Asp T (2015). Comparative transcriptome analysis within the *Lolium*/*Festuca* species complex reveals high sequence conservation. BMC Genom.

[64] He R, Kim MJ, Nelson W, Balbuena TS, Kim R, Kramer R (2012). Next-generation sequencing-based transcriptomic and proteomic analysis of the common reed, *Phragmites australis* (Poaceae), reveals genes involved in invasiveness and rhizome specificity. Am J Bot.

[65] Tian XJ, Long Y, Wang J, Zhang JW, Wang YY, Li WM (2015). De novo transcriptome assembly of common wild rice (*Oryza rufipogon* Griff.) and discovery of drought-response genes in root tissue based on transcriptomic data. PLoS ONE.

[66] Li M, Liang Z, Zeng Y, Jing Y, Wu K, Liang J (2016). De novo analysis of transcriptome reveals genes associated with leaf abscission in sugarcane (*Saccharum officinarum* L.). BMC Genom.

[67] Li HZ, Gao X, Li XY, Chen QJ, Dong J, Zhao WC (2013). Evaluation of assembly strategies using RNA-Seq data associated with grain development of wheat (*Triticum aestivum* L.). PLoS ONE.

[68] Krasileva KV, Buffalo V, Bailey P, Pearce S, Ayling S, Tabbita F (2013). Separating homeologs by phasing in the tetraploid wheat transcriptome. Genome Biol.

[69] Xu C, Jiao C, Zheng Y, Sun H, Liu W, Cai X, Dai S (2015). De novo and comparative transcriptome analysis of cultivated and wild spinach. Sci Rep.

[70] Farrell JD, Byrne S, Paina C, Asp T (2014). De novo assembly of the perennial ryegrass transcriptome using an RNA-Seq strategy. PLoS ONE.

[71] Fu N, Wang Q, Shen HL (2013). De novo assembly, gene annotation and marker development using Illumina paired-end transcriptome sequences in celery (*Apium graveolens* L.). PLoS ONE..

[72] Cardoso-Silva CB, Costa EA, Mancini MC, Balsalobre TWA, Canesin LEC, Rossini Pinto L (2014). De novo assembly and transcriptome analysis of contrasting sugarcane varieties. PLoS ONE.

[73] Kanehisa M, Araki M, Goto S, Hattori M, Hirakawa M, Itoh M (2008). KEGG for linking genomes to life and the environment. Nucleic Acids Res.

[74] Takagi H, Ishiga Y, Watanabe S, Konishi T, Egusa M, Akiyoshi N (2016). Allantoin, a stress-related purine metabolite, can activate jasmonate signaling in a MYC2-regulated and abscisic acid-dependent manner. J Exp Bot.

[75] Goyer A (2010). Thiamine in plants: aspects of its metabolism and functions. Phytochemistry.

[76] Rapala-Kozik M, Kowalska E, Ostrowska K (2008). Modulation of thiamine metabolism in *Zea mays* seedlings under conditions of abiotic stress. J Exp Bot.

[77] Zhong R, Ye ZH (2015). Secondary cell walls: biosynthesis, patterned deposition and transcriptional regulation. Plant Cell Physiol.

[78] Poorter H, Villar R, Bazzaz FA, Grace J (1997). The fate of acquired carbon in plants: chemical composition and construction costs. Resource allocation in plants.

[79] Wang J, Nayak S, Koch K, Ming R (2013). Carbon partitioning in sugarcane (*Saccharum* species). Front Plant Sci.

[80] Yoo CY, Hasegawa PM, Mickelbart MV (2011). Regulation of stomatal density by the GTL1 transcription factor for improving water use efficiency. Plant Signal Behav.

[81] Vij S, Tyagi AK (2006). Genome-wide analysis of the stress associated protein (SAP) gene family containing A20/AN1 zinc-finger (s) in rice and their phylogenetic relationship with *Arabidopsis*. Mol Gen Genom.

[82] Wang Y, Yang C, Jin Q, Zhou D, Wang S, Yu Y (2015). Genome-wide distribution comparative and composition analysis of the SSRs in Poaceae. BMC Genet.

[83] Liu Y, Zhang P, Song M, Hou J, Qing M, Wang W (2015). Transcriptome analysis and development of SSR molecular markers in *Glycyrrhiza uralensis* Fisch. PLoS ONE.

[84] Bose Mazumdar A, Chattopadhyay S (2016). Sequencing, de novo assembly, functional annotation and analysis of *Phyllanthus amarus* leaf transcriptome using the Illumina platform. Front Plant Sci.

[85] Mariani C, Cabrini R, Danin A, Piffanelli P, Fricano A, Gomarasca S (2010). Origin, diffusion and reproduction of the giant reed (*Arundo donax* L.): a promising weedy energy crop. Ann Appl Biol.

[86] Ahmad R, Liow PS, Spencer DF, Jasieniuk M (2008). Molecular evidence for a single genetic clone of invasive *Arundo donax* in the United States. Aquat Bot.

[87] Lawson MJ, Zhang L (2006). Distinct patterns of SSR distribution in the *Arabidopsis thaliana* and rice genomes. Genome Biol.

[88] Mun JH, Kim DJ, Choi HK, Gish J, Debellé F, Mudge J (2006). Distribution of microsatellites in the genome of *Medicago truncatula*: a resource of genetic markers that integrate genetic and physical maps. Genetics.

[89] Hong CP, Piao ZY, Kang TW, Batley J, Yang T, Hur Y (2007). Genomic distribution of simple sequence repeats in *Brassica rapa*. Mol Cells.

[90] Pramod S, Perkins AD, Welch ME (2014). Patterns of microsatellite evolution inferred from the *Helianthus annuus* (Asteraceae) transcriptome. J Genet.

[91] Levinson G, Gutman GA (1987). Slipped-strand mispairing: a major mechanism for DNA sequence evolution. Mol Biol Evol.

[92] King DG, Soller M, Kashi Y (1997). Evolutionary tuning knobs. Endeavour.

[93] Trifonov EN, Wasser SP (2004). Tuning function of tandemly repeating sequences: a molecular device for fast adaptation. Evolutionary theory and processes: modern horizons, papers in honour of Eviatar Nevo.

[94] Liu F, Hu Z, Liu W, Li J, Wang W, Liang Z (2016). Distribution, function and evolution characterization of microsatellite in *Sargassum thunbergii* (Fucales, Phaeophyta) transcriptome and their application in marker development. Sci Rep.

