# Supplementary material for: De novo assembly, functional annotation, and analysis of the giant reed (Arundo donax L.) leaf transcriptome provide tools for the development of a biofuel feedstock
Source: Biotechnol Biofuels. 2017 May 30;10:138. doi: 10.1186/s13068-017-0828-7 (PMC5450047; doi:10.1186/s13068-017-0828-7)
Supplement: Supplementary file 1 — Additional file 1. Description of water treatments imposition, soil water status, and meteorological conditions. [file 13068_2017_828_MOESM1_ESM.docx]

**Methods**

**Drought treatments and meteorological data**

Plants were irrigated until field capacity (θ_fc_) and then grown under two watering regimes during the 2014 dry season: well-watered (WW) and moderate drought stress (mDr). In both WW and mDr, θ_fc_ was 34%. The mDr was gradually imposed by decreasing the soil volumetric water content (θ) from 100% to approximately 40% of θ_fc_, while WW plants, used as controls, were maintained above 80% of θ_fc_ with controlled drip-irrigation to replenish evapotranspiration losses. θ was continuously checked in the two treatments using a time domain reflectometry SM150 Soil Moisture Sensor (Delta-T Devices Ltd., Cambridge, UK). One soil moisture sensor per treatment was placed at 50 cm depth to measure daily θ. Four measures per day were recorded using a DL6 Data Logger (Delta-T Devices Ltd., Cambridge, UK), and the daily average θ was calculated. Additionally, daily water deficit (Wd) was calculated as the difference between water availability (i.e. rainfall) and crop water demand (i.e. specific crop evapotranspiration). Meteorological data have been recorded and stored using a weather station installed close to the experimental crop field to monitor air temperature and rainfall.

**Results**

**Soil water status and meteorological conditions**

During the experimental period, from June 24 (DOY 175) to September 5 (DOY 248), the mean average air temperature was 20.4 °C, the mean maximum and minimum air temperature were 25.7°C and 15.6°C, respectively. At T1 (DOY 198), air temperature rose from a minimum of 14.9°C to a maximum of 30.4°C; similarly, at T2 (DOY 218), air temperatures increased from a minimum of 15.1°C to a maximum of 27.4°C, and at T3 (DOY 248), air temperatures changed from a minimum of 14.5 °C to a maximum of 25.2 °C (Additional file 2: Fig. S1 a).

A total rainfall of 147 mm provided an insufficient amount of water to replenish the 270 mm of total Wd in mDr condition (Additional file 2: Fig. S1 b). Consequently, at T1, the cumulated Wd from the beginning of water withhold was estimated to be 93.7 mm, and cumulated Wd of 147.1 mm and 270 mm were observed at T2 and T3, respectively (Additional file 2: Fig. S1 b). In addition, an average daily Wd of 3.7 mm was caused by an average daily crop evapotranspiration of 5.3 mm.

Finally, the progressive decrease of θ, expressed as percentage of the θ_fc_ (equal to 34% and corresponding to a 100% of evapotranspiration restitution), observed in mDr in response to water limitation is reported in Additional file 2: Fig. S1 c. After water withholding different trends of θ, particularly from DOY 192 were shown between the two treatments. Then, this difference increased from 23.1% at T1 to 37.7% at T2, and finally to 38.5% at T3. Throughout the experiment, θ was maintained above the 80% of θ_fc_ in WW, whereas in mDr, θ reached 39.8% of θ_fc_.
