# Supplementary figures and images for: De novo assembly, functional annotation, and analysis of the giant reed (Arundo donax L.) leaf transcriptome provide tools for the development of a biofuel feedstock
Source: Biotechnol Biofuels. 2017 May 30;10:138. doi: 10.1186/s13068-017-0828-7 (PMC5450047; doi:10.1186/s13068-017-0828-7)

## Slide 1
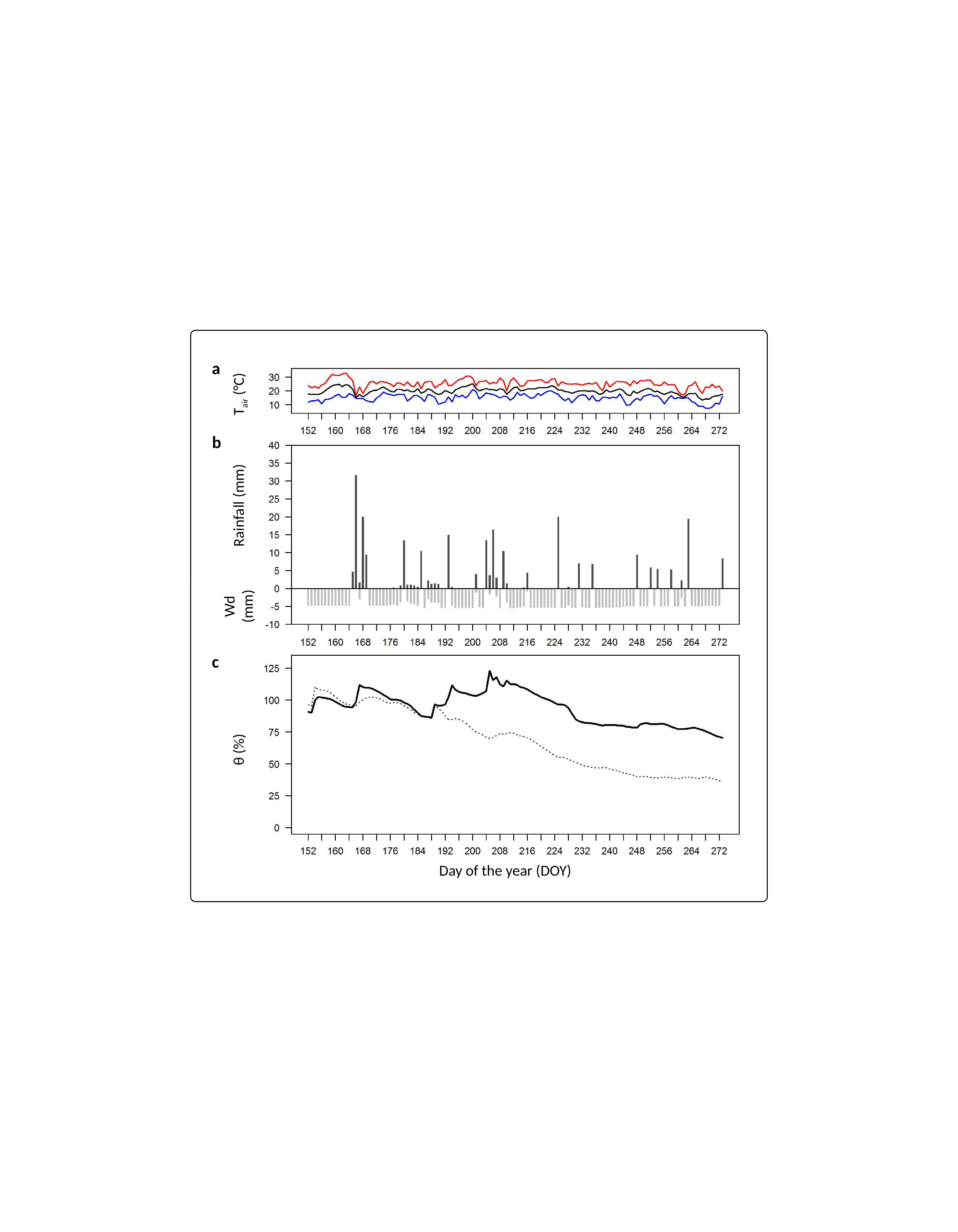

a
Tair (°C)
b
Rainfall (mm)
Wd (mm)
c
θ (%)
Day of the year (DOY)

Supplement: Supplementary file 2 — Additional file 2: Figure S1. Soil water status and meteorological conditions at the experimental site in 2014 observed from DOY 152 to DOY 272. (a) Daily maximum (red line), mean (black line), and minimum (blue line) air temperature (Tair, °C) are shown. (b) Daily rainfall (Rainfall, mm) and water deficit (Wd, mm) are shown as black and gray bars, respectively. The positive values of Wd are not reported. (c) Soil volumetric water content (θ), expressed as percentage of the field capacity (θfc), is shown as black solid and black dashed lines in WW and in mDr, respectively. [file 13068_2017_828_MOESM2_ESM.pptx]

## Slide 1
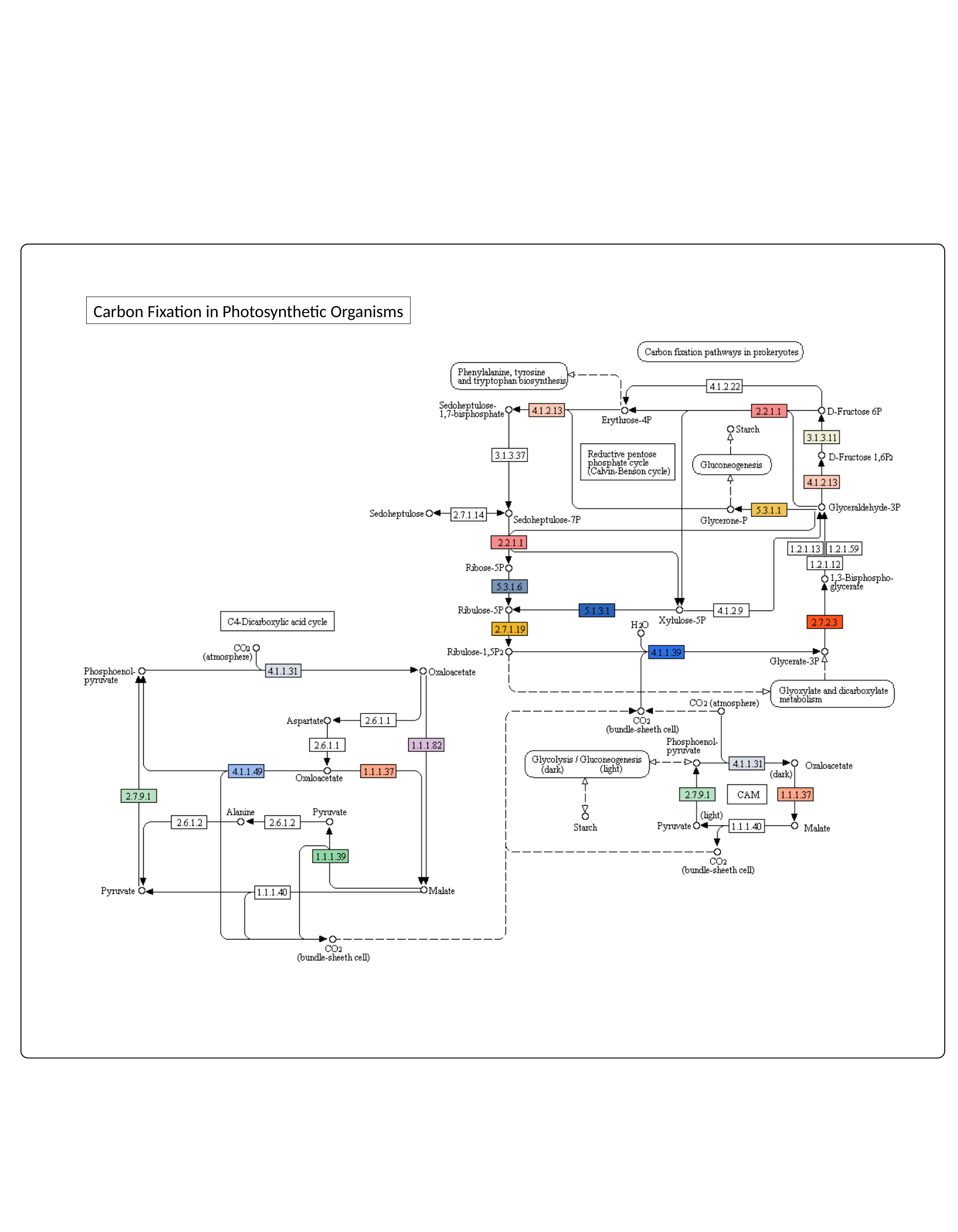

Carbon Fixation in Photosynthetic Organisms

Supplement: Supplementary file 5 — Additional file 5: Figure S2. Carbon fixation pathway genes found in A. donax leaf transcriptome are depicted by the different colored ECs (one color for each EC). [file 13068_2017_828_MOESM5_ESM.pptx]

## Slide 1
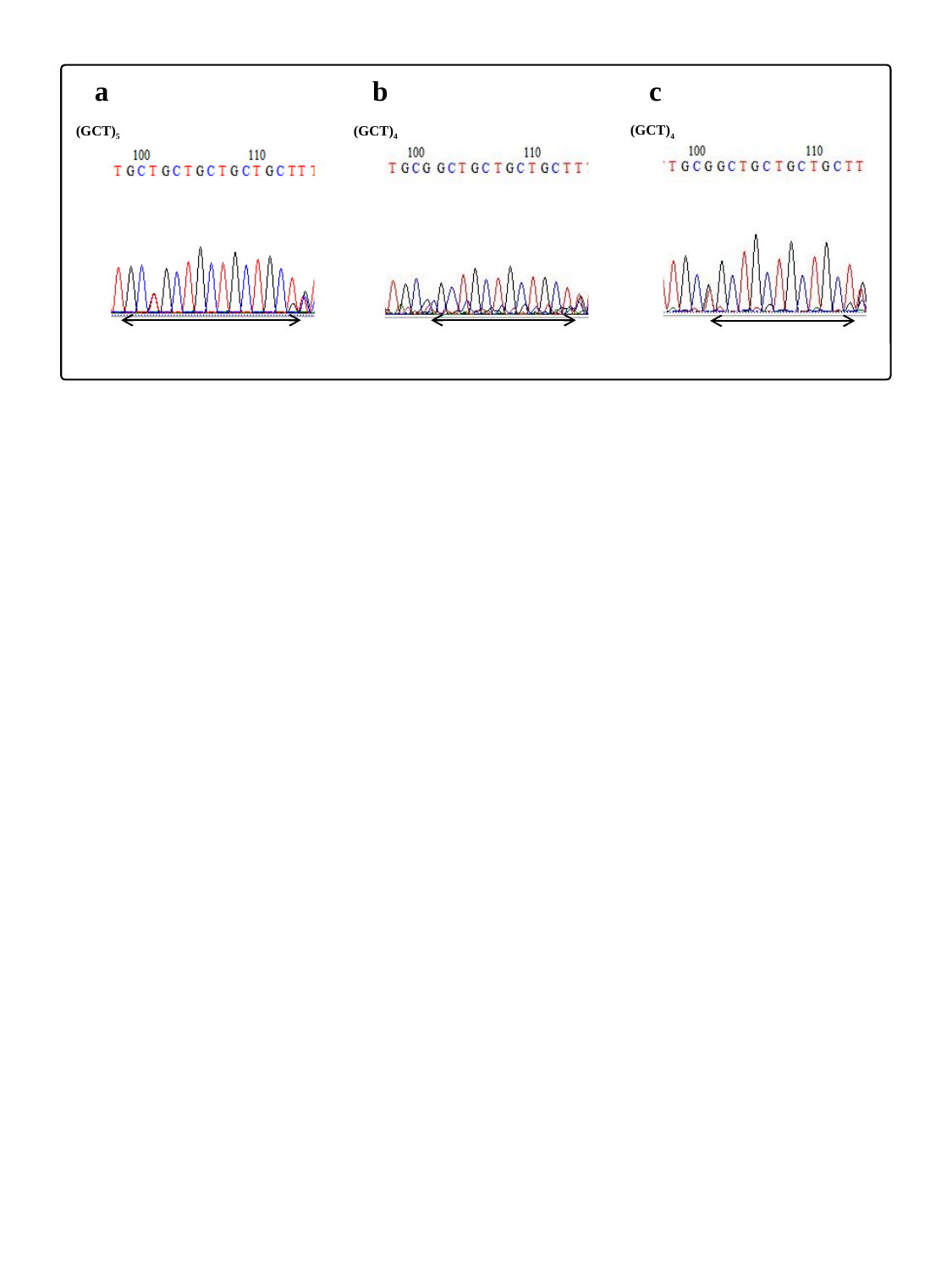

a
b
c
(GCT)4
(GCT)5
(GCT)4

Supplement: Supplementary file 10 — Additional file 10: Figure S3. Electropherogram analysis of the CPSSR_4 PolySSR in the three A. donax ecotypes. Electropherograms of the sequenced fragments of EcoA (a), EcoB (b) and EcoC (c). [file 13068_2017_828_MOESM10_ESM.pptx]
